# Assessing the Control of Preservational Environment on Taphonomic and Ecological Patterns in an Oligocene Mammal Fauna from Badlands National Park, South Dakota

**DOI:** 10.1371/journal.pone.0157585

**Published:** 2016-06-15

**Authors:** Paige K. Wilson, Jason R. Moore

**Affiliations:** 1 Department of Earth Sciences, Dartmouth College, Hanover, New Hampshire, United States of America; 2 Current Affiliation: Honors College, University of New Mexico, Albuquerque, New Mexico, United States of America; New York Institute of Technology, UNITED STATES

## Abstract

Comparisons of paleofaunas from different facies are often hampered by the uncertainty in the variation of taphonomic processes biasing the paleoecological parameters of interest. By examining the taphonomic patterns exhibited by different facies in the same stratigraphic interval and area, it is possible to quantify this variation, and assess inter-facies comparability. The fossil assemblages preserved in Badlands National Park (BNP), South Dakota, have long been a rich source for mammalian faunas of the White River Group. To investigate the influence of the variation of taphonomic bias with lithology whilst controlling for the influence of changes in patterns of taphonomic modification with time, taphonomic and paleoecological data were collected from four mammal-dominated fossil assemblages (two siltstone hosted and two sandstone hosted) from a narrow stratigraphic interval within the Oligocene Poleslide Member of the Brule Formation, in the Palmer Creek Unit of BNP. Previous work in the region confirmed that the two major lithologies represent primarily aeolian- and primarily fluvial-dominated depositional environments, respectively. A suite of quantifiable taphonomic and ecological variables was recorded for each of the more than 800 vertebrate specimens studied here (857 specimens were studied in the field, 9 specimens were collected and are reposited at BNP). Distinctly different patterns of taphonomic biasing were observed between the aeolian and fluvial samples, albeit with some variability between all four sites. Fluvial samples were more heavily weathered and abraded, but also contained fewer large taxa and fewer tooth-bearing elements. No quantifiable paleofaunal differences in generic richness or evenness were observed between the respective facies. This suggests that while large vertebrate taxonomic composition in the region did vary with paleodepositional environment, there is no evidence of confounding variation in faunal structure, and therefore differences between the assemblages are attributed to differing preservational environments producing a taphonomic overprint on the assemblages. The lack of apparent taphonomic bias on paleofaunal structure suggests that such paleoecological data can be compared throughout the Poleslide Member, irrespective of lithology.

## Introduction

Taphonomic biasing is one of the major obstacles impeding paleoecological reconstruction. Any alteration to the taxa represented in a fossil collection masks potential inventory of those members [1–5]. Statistically significant differences in assemblage membership can therefore either be attributed to preservational bias or to true ecological differences in the fauna [2,6]. Therefore, in order to analyze any paleoecological dataset, we must first understand the post-mortem modifications that may have affected that dataset. In this study we assess both the taphonomic biasing and the faunal structure of assemblages from two distinct depositional environments within the same formation to better understand how preservational environment influences paleoecological signal fidelity.

**Fig 1:**
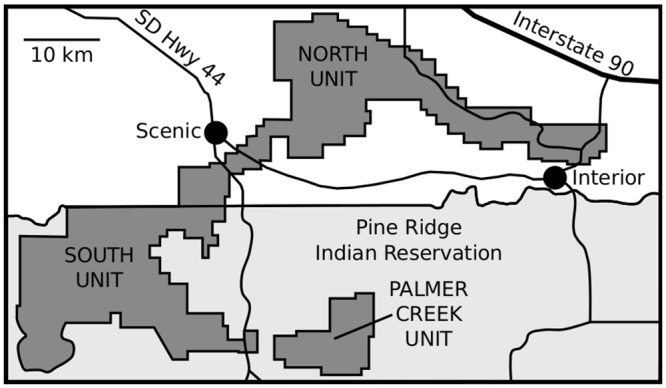
Map of Badlands National Park. Map of Badlands National Park with the Palmer Creek Unit highlighted.

The White River Group (WRG) of central North America provides an unrivalled opportunity to study vertebrate faunal structure and dynamics during the latter portion of the Paleogene. The terrestrial sediments of the WRG are extremely fossiliferous, preserving abundant mammal, reptile, and bird fossils from a period spanning ~36.5 Ma to ~30 Ma [7]. The WRG has a long history of paleontological study, particularly in Badlands National Park [8–12]. Initial study of the WRG in the area of Badlands National Park drew on outcrops from all areas now administered by both the National Park Service and the Oglala Sioux Tribe, and noted some significant differences in lithology and fossil composition within formations and members of the WRG in different regions of the park [13]. Recent study has, however, focused on exposures in the North Unit of Badlands National Park, over the equally, if not more significant exposures in the South Unit of Badlands National Park. In this study, courtesy of the collaboration of the Oglala Sioux Tribe and the Oglala Sioux Parks and Recreation Department, we were able to undertake the first significant paleontological survey of an area of the South Unit in more than 30 years, focusing on sections within the Palmer Creek Unit (Fig 1).

The area of the Palmer Creek Unit that was surveyed contains the Oligocene Poleslide Member of the Brule Formation [14], a unit that is dated between ~32 and ~30 Ma, and spans the late Orellan and early Whitneyan North American Land Mammal Ages [7]. In the Palmer Creek Unit, unlike in the North Unit, the Poleslide Member is composed of alternating ribbon sandstone deposits (coarse-grained, lenticular bodies, fining upwards and exhibiting internal trough cross-bedding, preserving fish and freshwater bivalves) and massive siltstone deposits (little evidence of any internal primary sedimentary structures, albeit with some pedogenic overprinting), interpreted as small-scale meandering channels cutting through primarily loessic floodplain sediments. Abundant vertebrate fossils are preserved in both facies from the same stratigraphic levels, hence sampling as nearly as possible from the same fauna. Studying assemblages collected from these facies provides the ideal opportunity to test the influence of preservational environment on both the patterns of taphonomic modification undergone by an assemblage, and on the ecological signals from the fauna that are preserved.

The issue of inter-environmental variability in taphonomic bias obfuscating potential paleoecological signals is perennial in paleontology. It was recognized during the beginnings of the scientific practice of paleontology [15], and was documented by Efremov [16] during his definition of the branch of taphonomy. Detailed studies of marine invertebrate assemblages demonstrated that patterns of taphonomic modification correlate with facies of preservation in many instances [17–19] leading to the concept of “taphofacies.” It is less certain to what extent such varying taphonomic modification influences parameters of paleoecological interest in these invertebrate assemblages, although the severity of biasing will vary depending on the particular considered biological parameter. There has been less study of the influence of preservational environment on taphonomic pattern in terrestrial vertebrate assemblages, despite the abundant literature investigating and describing the actions of individual taphonomic biasing agents [20–26]. A recent meta-analysis [27] has suggested that there is surprisingly little correlation between preservational environment and taphonomic pattern in a set of large vertebrate accumulations, however these data were collected by a number of investigators from a wide range of time periods and geographic locations. Consequently studies with greater control are required to assess such relationships independent of all of such confounding factors.

With a clear understanding of the variation of taphonomic biasing among closely related terrestrial assemblages preserved in different facies, it becomes possible to address questions of broader paleoecological importance. Most crucially among these is the degree of paleoenvironmental (and hence likely taphonomic) similarity that is required in order to safely compare paleoecological parameters among terrestrial vertebrate fossil assemblages. If taphonomic modification either varies little between environments of preservation, or paleoecological parameters are not significantly influenced by variation in taphonomic modification, then a wide range of paleoecological comparisons become possible among disparate terrestrial vertebrate assemblages. Alternatively, if taphonomic modification varies significantly between environments of preservation and leads to bias in paleoecological parameters of interest, a much more measured strategy for assessing faunal paleoecology is required (e.g., that suggested by Moore and Norman [26]).

To begin to address these questions, we compare taphonomic data from two sets of fossil assemblages collected at the same time, by the same researchers from two different facies (aeolian siltstones vs. fluvial sandstones) within the Poleslide Member of the Brule Formation in the Palmer Creek Unit of Badlands National Park. We then assess the similarities in species richness, species evenness, and faunal composition among the sampled assemblages in light of the observed taphonomic differences associated with each facies to determine the influence of preservational environment on the biasing of these paleoecological parameters.

## Materials and Methods

The paleontological surveys of the Palmer Creek Unit were undertaken under US National Park Service Permit #BADL-20110SCI-0004, and access for the surveys was with special permission of the Oglala Sioux Parks and Recreation Authority. All necessary permits were obtained for the described study, which complied with all relevant regulations. In accordance with the terms of the permit, extensive fossil collection from the Palmer Creek Unit was not possible, therefore all specimens (842 in total) were described in the field and extensively photographed with scale bars to permit later verification of field collected data. A limited number of scientifically exceptional specimens (9) were collected and are currently reposited at Badlands National Park (Specimen numbers: BADL 59763–59771). See S1 Appendix for full dataset.

Four fossiliferous localities were identified and studied within a 10 meter stratigraphic thickness of the Poleslide Member of the Brule Formation in the Palmer Creek Unit of Badlands National Park. The four localities were separated by a maximum of 500 meters. All localities were on land administered jointly by the US National Park Service and the Oglala Sioux Parks and Recreation Authority—locality information is available through the Badlands National Park Paleontologist. Two of the localities (A and B) were overbank siltstones, whereas the other two (C and D) represent channel sandstones.

The surveyed area of the Poleslide Member contains a number of lenticular, single-story channel bodies, 2 meters deep and up to 10s of meters in width. Individual channels can be traced for hundreds of meters parallel to paleoflow. The sandstones of these channel bodies are green in color and are generally coarsely grained; grain size ranges from medium-grained sands to granules, with an average of very coarse grained sand. The lower portion of the channel bodies are coarser-grained and show abundant centimeter-scale trough cross-bedding. The channel bodies fine upwards, and centimeter-scale current ripples become more common. The presence of articulated unionid bivalves in some of the channel deposits indicates fast-moving, well-oxygenated water. Two separate channels were sampled for this study, both of which show fossil preservation throughout the thickness of the channel deposit.

Homogeneous tan siltstone horizons are interbedded with the channel sandstones in this portion of the Poleslide Member. These horizons can be upwards of 3 meters thick, and display a uniform grain-size throughout and few recognizable sedimentary structures. There is limited evidence of post-depositional modification of these siltstones by soil forming processes, although scattered horizons of pedotubules, interpreted as root traces, are present. These soil forming processes can be observed elsewhere in the Poleslide as well. These units are interpreted to represent deposits of loess accumulating between the Poleslide channels, potentially overprinted by pedogenic processes, or remobilized by occasional, low-energy sheet flows, although such flows would have to be sufficiently low energy not to leave scours or other sedimentary structures. This agrees with previous interpretations of loessic genesis for the Poleslide siltstones elsewhere in the WRG [28]. Two separate sections of Poleslide siltstone were sampled as part of this study, and each showed preservation of fossil vertebrates throughout the unit thickness.

Approximately 250 terrestrial vertebrate specimens were examined from each of the four localities. Protocols were designed to avoid collector bias in terms of size and identification; all fragments that were identifiable either to a taxonomic level more detailed than class, or to skeletal element were recorded. For each locality, all floating and *in situ* specimens were examined from a ~2 meter stratigraphic thickness, across 20 to 250 horizontal meters of outcrop. For the channel-hosted localities this corresponded to a single fossiliferous sandstone unit, for the siltstone localities, however, observed *in situ* specimens were spread evenly throughout the recorded intervals. Areas where downslope movement of fossils or sediment had introduced specimens from higher levels were avoided to minimize time-averaging in each of the samples.

To enable the taphonomic and ecologic comparison of the four assemblages, a suite of eleven characteristics were measured from each sampled specimen. These included skeletal element and taxon identification, and specimen maximum size (in mm), weathering (stages 0–3, after Fiorillo [3]), and abrasion (stages 0–3, after Fiorillo [3]). Using the methods developed by Moore and Norman [26], columnarity, flatness, density, and surface area to volume ratios were assigned to each specimen based on element classification. Details for the values for each of these characteristics assigned to each element category are given by Moore and Norman [26]. These metrics were determined for the complete element to which each specimen belonged based on existing measurements. Element bulk bone densities were calculated from the average of those presented by Behrensmeyer (1975). Surface area to volume ratio was assessed on a coarse, five-point scale, assigned by element [26], as the assessment of bone surface area is very difficult. Elements were also classified into Voorhies Groups [29], which provide an approximation of the transportability of bones in a fluvial environment, with Voorhies Group I elements being the most easily transported elements, and Group III elements the least.

Specimens were identified to the genus level wherever possible–consistent species level identifications were not possible from field and photographic observations. For teeth and complete diagnostic elements, genus-level identifications were accurate and consistent between the field and later analysis of photographs. A limited number of potentially diagnostic incomplete elements could not be identified in the field or from the photographs (approximately 50). If patterns of breakage differ among samples, this variation in identifiability will introduce an additional source of error to the measured taxon abundance distributions. To account for the error introduced by differing brakeage patterns, each specimen was also given a “size category” in an attempt to recover further ecological and taphonomic patterns from the dataset. These size categories ranged from 1 to 6, based on published adult body size estimates for the constituent taxa ([30], Table 1). These categories are assigned to differentiate the most common taxa in WRG assemblages (small rodents; large rodents and lagomorphs; hypertragulids and leptomerycids; oreodonts, horses, camels and hyracodonts; rhinocerotids). Elements not identifiable to taxon were assigned to the size category of taxa with comparably sized identifiable elements. In an attempt to further test the extent of identification biases, analyses were re-run with abundances solely from dental elements, which should exhibit the minimum bias among samples. The complete dataset was rigorously tested, and does not violate the assumptions of multiple regression (normality, heteroskedasticity, autocorrelation and collinearity).

**Table 1:** Definition of Size Categories.

Category	Example Taxa	Adult Body Mass
1	*Eumys*	<0.5 kg
2	*Paleolagus*	0.5–5 kg
3	*Hypertragulus*	5–40 kg
4	*Merycoidodon*	40–100 kg
5	*Subhyracodon*	>100 kg

Size categories with example taxa and estimated adult body mass [30].

Two sets of analyses were undertaken using the collected dataset. The first was aimed at analyzing taphonomic patterns at each of the four sites, to quantify the post-mortem biases that affected the assemblages. It is important to consider both how the individual measured taphonomic characteristics vary between sampled facies, but also how the total pattern of modification differs, and therefore taphonomic bias was quantified in two manners. Firstly, univariate comparisons of individual measured characteristics were undertaken among localities. Note that univariate analyses of patterns of density, flatness, columnarity, and surface area to volume ratio were not undertaken as these are secondary characteristics derived from the identity of each collected element. Consequently gross patterns of variation within these characteristics are expected to be reflected in the observed variation of skeletal element abundances. The frequency distributions of the different taphonomic characteristics were tested against the null hypothesis that they all belonged to the same distribution using *G*-tests [31].

Secondly, multiple regression analyses of taphonomic pattern were undertaken to examine the similarity of total taphonomic pattern among assemblages (following Moore and Norman [26]). These analyses simultaneously quantify the influence, or lack thereof, of all of the measured characteristics (independent variables) on %MAU (the represented percentage of the “minimum animal unit” [32]; dependent variable)–a measure of total taphonomic biasing which quantifies the proportional under- or overrepresentation of each skeletal element in an assemblage in comparison to the proportion expected in a complete skeleton. %MAU ranges from zero to 100 for underrepresented elements (i.e. <100% of the expected abundance of that particular element in the assemblage), and is greater than 100 for overrepresented elements. The optimum regression model is chosen by stepwise model selection removing non-significant taphonomic characters until the model with the lowest AIC value (*i*.*e*., the most informative with the lowest complexity [33]) is reached [31]. To minimize the overrepresentation of elements that are easily identified from incomplete specimens, tooth fragments were excluded from these calculations. Multivariate analysis of variance (MANOVA) was used to test the similarity of the multiple regression-based taphonomic models among all four sites (by simultaneously comparing the distributions of the statistically important taphonomic characteristics), and, more generally, to test the similarity of sandstone facies to siltstone facies sites.

With the taphonomic bias at each site quantified, the assemblages were compared in terms of faunal composition and diversity. Faunal diversity variation among localities was assessed utilizing Shannon entropy, true diversity and rarefaction techniques to capture variations in both richness and evenness [34]. A simple measure of this is Margalef’s richness index ((*S*-1)/ln(*n*), where S = number of species and *n* = number of individuals in the sample [35]. Shannon entropy [36], also known as the Shannon or Shannon-Wiener index, is a diversity metric that quantifies the difficulty in predicting the taxonomic identity of a random individual drawn from a chosen population. True diversity is the number of equally common species that will give a value of a chosen diversity metric equal to that measured from a sample [37].

Differences in Shannon entropy among samples (either within a single facies, or between facies) were tested by the calculation of simultaneous confidence intervals using bootstrap methods, following Scherer et al. [2013] [38]. Differences in genus proportional abundances were also assessed to determine whether the abundance distributions of samples within and among facies were similar. Differences in proportional abundances could be present in assemblages with similar diversities and evennesses.

Comparison of the results of the taphonomic and paleoecological analyses could produce one of four potential outcomes, each with different implications for the interpretation of the Palmer Creek assemblages (summarized in Table 2).

**Table 2:** Potential Implications of Comparisons Between Taphonomic and Ecological Analyses.

	***Facies ecologically similar***	***Facies ecologically different***
***Facies taphonomically similar***	Facies of preservation does not affect taphonomic history. Landscape-scale paleoenvironmental preferences of Palmer Creek taxa not captured, or similar.	Facies of preservation does not affect taphonomic history. Landscape-scale paleoenvironmental preferences of Palmer Creek taxa captured.
***Facies taphonomically different***	Facies of preservation influences taphonomic history. Landscape-scale paleoenvironmental preferences of Palmer Creek taxa likely similar.	Facies of preservation influences taphonomic history. Landscape-scale paleoenvironmental preferences of Palmer Creek taxa cannot be interpreted.

All statistical analyses were carried out in R 3.1.1 (R Development Core Team 2014) using the packages *Biodiversity*, *vegan*, *simboot* and *stats* [39, 40, 41].

## Results

### Comparison of Taphonomic Modification

#### Element Size-Frequency Distribution

**Fig 2:**
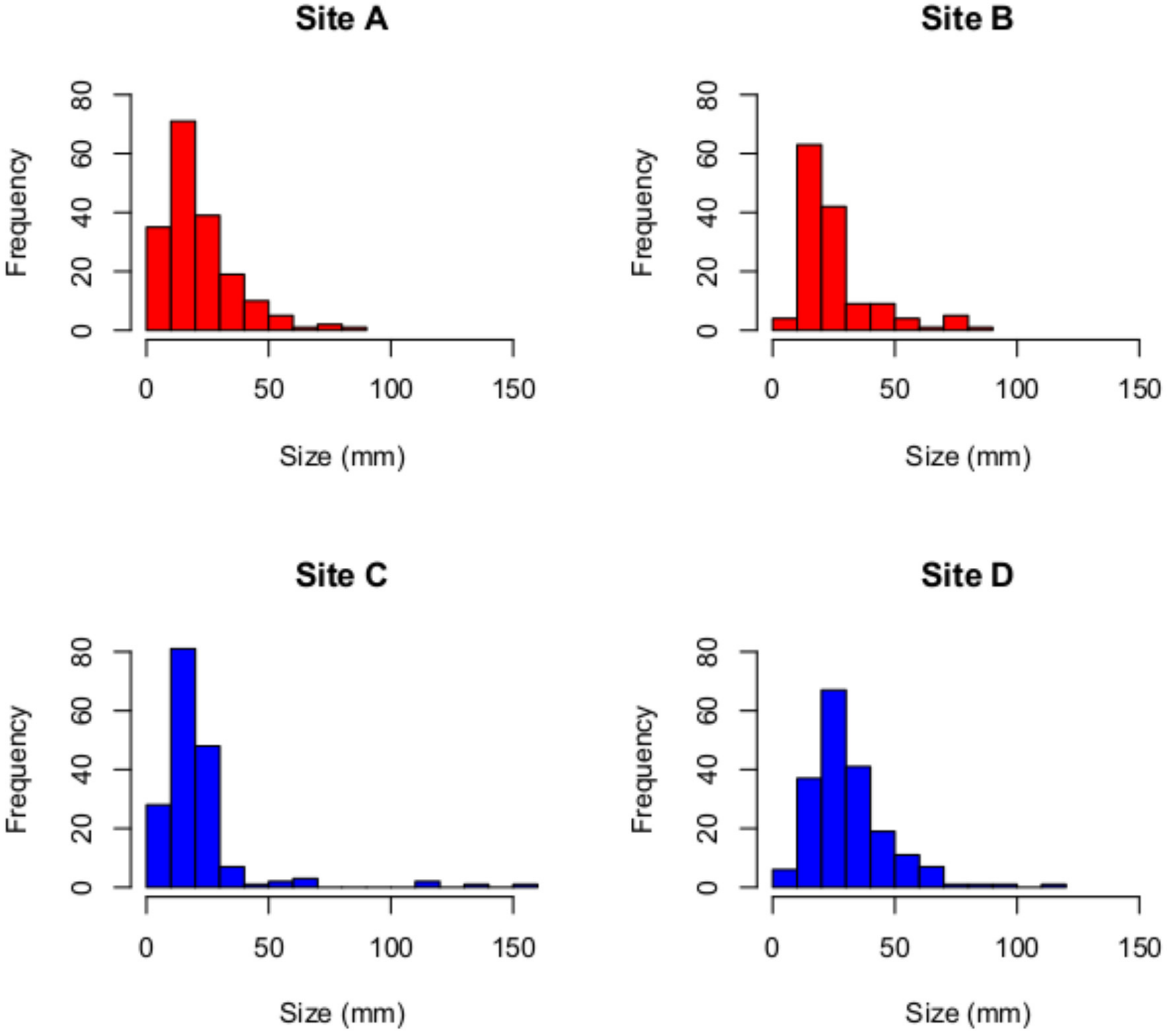
Distribution of Element Size. Histogram of size (mm) of elements at each of the four study sites; no significant differences between the sites indicated.

Measured element sizes ranged from 1 mm to 160 mm, with each of the four studied localities averaging between 21 and 32 mm (Fig 2). Pooled size-frequency distributions for each facies are identical (*G*-test, *p* = 0.144, binned at 5 mm increments with all specimens over 70mm combined into a single bin).

#### Weathering Stage

The majority of elements recovered from all four localities exhibited little weathering, although elements showing weathering stage 1 were the most common (Table 3). There is a statistically significant difference between pooled weathering stage distributions for each facies (*G*-test, *p* = 0.006). The sandstone-hosted assemblages (C and D) show a greater proportion of more strongly weathered elements than the siltstone-hosted assemblages (A and B). Weathering stage distributions also differ within each facies (siltstone and sandstone: *G*-test, *p* < 0.001).

**Table 3:** Weathering Stage Distribution.

Weathering Stage	Site A	Site B	Site C	Site D
0	108	32	102	40
1	65	96	58	117
2	9	10	12	34
3	0	0	2	1

Weathering stages are based on Fiorillo [3].

#### Abrasion Stage

The majority of studied skeletal elements (508/686) showed no evidence of abrasion at all (Table 4). Comparison of abrasion stage distribution between facies shows a statistically significant difference (*G*-test, *p* = 0.004). Again, it is the sandstone-hosted assemblages (C and D) that show an increased proportion of more strongly abraded elements over the siltstone-hosted assemblages (A and B). Abrasion stage distributions are not significantly different within facies (siltstone: *G*-test, *p* = 0.18, sandtone: *G*-test, *p* = 0.11).

**Table 4:** Abrasion Stage Distribution.

Abrasion Stage	Site A	Site B	Site C	Site D
0	140	114	127	127
1	36	23	33	55
2	6	1	11	10
3	0	0	3	0

Abrasion stages are based on Fiorillo [3].

#### Skeletal Element Abundances

**Fig 3:**
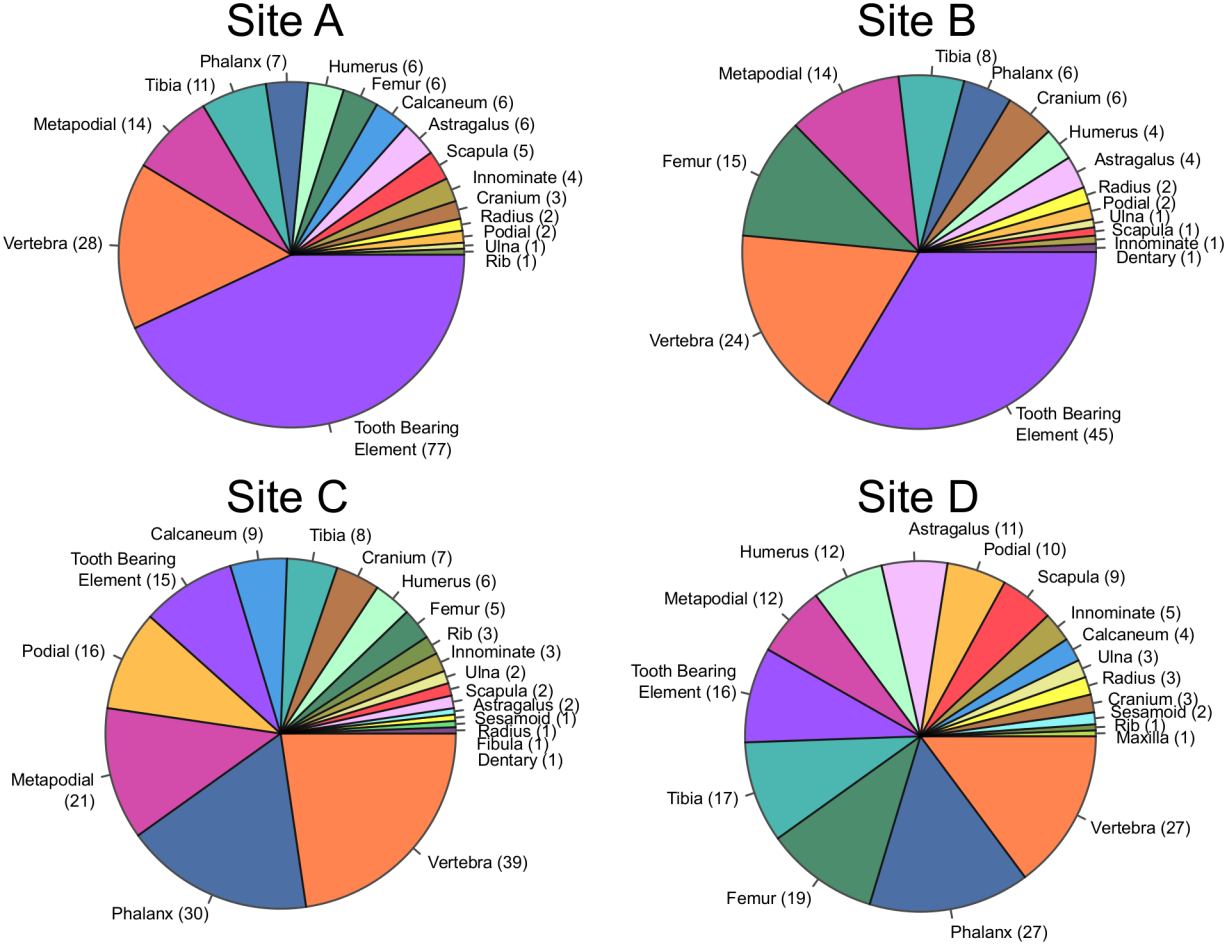
Distribution of Elements by Facies. The distribution of elements at the sandstone and siltstone sites is notably different in the proportion of tooth bearing elements; sandstone sites appear to be dominated by this category, while siltstone sites are not.

Even if the physical characteristics of the skeletal elements preserved at each locality remain the same, differences in element identity could be present. Comparison of element abundances at each site shows statistically significant differences in distribution between facies (*G*-test, *p* < 0.001; Fig 3). Interestingly, the vast majority of this difference in element abundances comes from the significant increase in the abundance of tooth-bearing elements in siltstone-hosted assemblages. Skeletal element abundance distributions are not statistically different between the siltstone assemblages (*G*-test, *p* = 0.51), but are different between the sandstone assemblages (*G*-test, *p* < 0.001).

A comparison of the abundance of Voorhies Group [29] I vs. III elements (i.e. rib, vertebra, scapula and sternum vs. skull and mandible) between the two facies shows a significantly greater abundance of Group I elements (those that are more easily transported) in the sandstone sites, and a greater abundance of Group III elements (those that are less easily transported) in the siltstone sites (*G*-test, *p* < 0.001). Voorhies Group I:III ratios do not differ within facies (siltstone: *G*-test, *p* = 0.80, sandtone: *G*-test, *p* = 0.93).

#### Taxon Size Distribution

The previously discussed taphonomic characteristics do not directly address variation in preservation potential between taxa of different sizes. There are significant differences in taxon size class distribution between facies (*G*-test, *p* < 0.001; Table 5), with the siltstone-hosted assemblages containing a greater proportion of smaller taxa than the sandstone-hosted assemblages. Taxon size class distributions are statistically significantly between localities within each facies (siltstone: *G*-test, *p* < 0.001, sandtone: *G*-test, *p* = 0.009).

**Table 5:** Taxon Size Class Distribution.

Size Category	Site A	Site B	Site C	Site D
1	5	0	3	1
2	29	5	2	0
3	50	20	7	26
4	59	84	12	83
5	37	23	6	69

Size categories are defined in Table 1.

#### Overall Taphonomic Pattern

Combining all collected taphonomic characteristics into a multivariate dataset provides the opportunity of a more nuanced analysis and interpretation of the factors influencing the pattern of taphonomic modification of an assemblage by accounting for the interactions among characteristics, rather than simply individual patterns.

Multiple regression analysis comparing the observed taphonomic characteristics to the relative over- or underrepresentation of different skeletal elements (Table 6; see Moore and Norman [26] for complete details of the method) shows the most significant factors in determining overall pattern of skeletal element abundance at all four localities to be element density and element columnarity (*p* < 0.001). Columnarity and density also show similar regression coefficient magnitudes across three of the four sites. The one exception is in Site D, where columnarity appears positively correlated to element abundance bias, rather than negatively correlated as at the other sites. Surface area to volume ratio (SA:V) also appears to be related to the likelihood of element preservation at most sites (Table 6). Abrasion, element size, and taxon size category were not significantly correlated to element representation at any of the studied localities, while weathering shows a weak correlation at a single site (B; Table 6).

**Table 6:** Results of Multivariate Regression Analysis of Taphonomic Variables.

	Site A (0.92)	Site B (0.92)	Site C (0.91)	Site D (0.58)
Intercept	**-11020.00**	**-7801.85**	-878.59	-677.10
Weathering	-30.40	**126.98**	35.62	49.60
Abrasion	-114.10	-77.36	-6.75	22.91
Size (mm)	0.29	-1.71	-0.21	2.76
Size Category	-46.40	3.41	-2.31	-39.65
Density	**6427.00**	**5333.05**	**1166.61**	**877.46**
Flatness	**-1081.00**	63.23	1273.04	-241.96
Columnarity	**-1801.00**	**-1155.30**	**-595.35**	**474.49**
SA:V	**214.70**	33.04	**143.13**	**-63.09**
*F*-score	249.6	188.7	36.34	30.53
*DF*	166.00	121.00	20.00	163.00

Values are the regression coefficients calculated for each variable, those in bold are statistically significant (*p* < 0.05). *R*^*2*^ values recorded in parentheses next to each site. Degrees of freedom and *F*-score reported at bottom. Sites A and B were sandstone hosted, sites C and D were siltstone hosted.

MANOVA analysis (Table 7) comparing the overall taphonomic patterns (*i*.*e*., the complete model including all nine taphonomic variables), and the subset of statistically significant characteristics from the prior regression analyses, indicates that some of the four sites show statistically significantly different patterns of taphonomic modification (*p* < 0.001). Analyzing pairs of localities separately shows that the taphonomic pattern of every pair is statistically distinct (MANOVA, *p* < 0.001), except for the pair of sandstone-hosted localities when only considering the subset of taphonomic characteristics controlling element representation bias.

**Table 7:** Summary of *p*-values Reported from MANOVA.

	All Factors	Density, Flatness, Columnarity, and SA: V
A, B, C, D	<0.001	<0.001
Sandstone	<0.001	0.1852
Siltstone	<0.001	0.0021

The category “All Factors” includes weathering, abrasion, size, size category, density, flatness, columnarity, SA:V, and %MAU. The second category includes only density, flatness, columnarity, and SA:V. The first test compares all four sites, the second compares Site A against B, and the third compares Site C against D.

In summary, the overall taphonomic patterns of siltstone-hosted assemblages were significantly different from those of sandstone-hosted assemblages. Intra-facies comparisons of taphonomic pattern were much more similar that inter-facies patterns, although small, but statistically significant differences in taphonomic pattern were present between the two sandstone-hosted assemblages.

### Comparison of Ecological Structure

Taxonomic diversity does not differ significantly among the four sampled localities. This can be illustrated by calculating and comparing Shannon entropy for the raw sampled data at each locality (Table 8). Comparison of the Shannon entropy between siltstone and sandstone sites (*p* = 0.63)failed to show any statistically significant differences in genus diversity [42]. Similar patterns were observed when examining only dental elements to examine the extent of identification bias; only results from the larger dataset are reported here, for clarity.

**Table 8:** Diversity and richness at Sites A, B, C, and D.

	Site A	Site B	Site C	Site D
Shannon-Wiener	2.290	1.688	1.881	2.112
Rarefied Richness (*n* = 20)	9.304	8.206	7.805	11.000
Margalef's Richness Index	2.772	2.405	2.272	3.338

All sites rarefied to 20 specimens. Results appeared similar regardless of index; Shannon-Wiener, Rarefied Richness, and Margalef’s Richness Index are given as typical of the assemblages.

**Fig 4:**
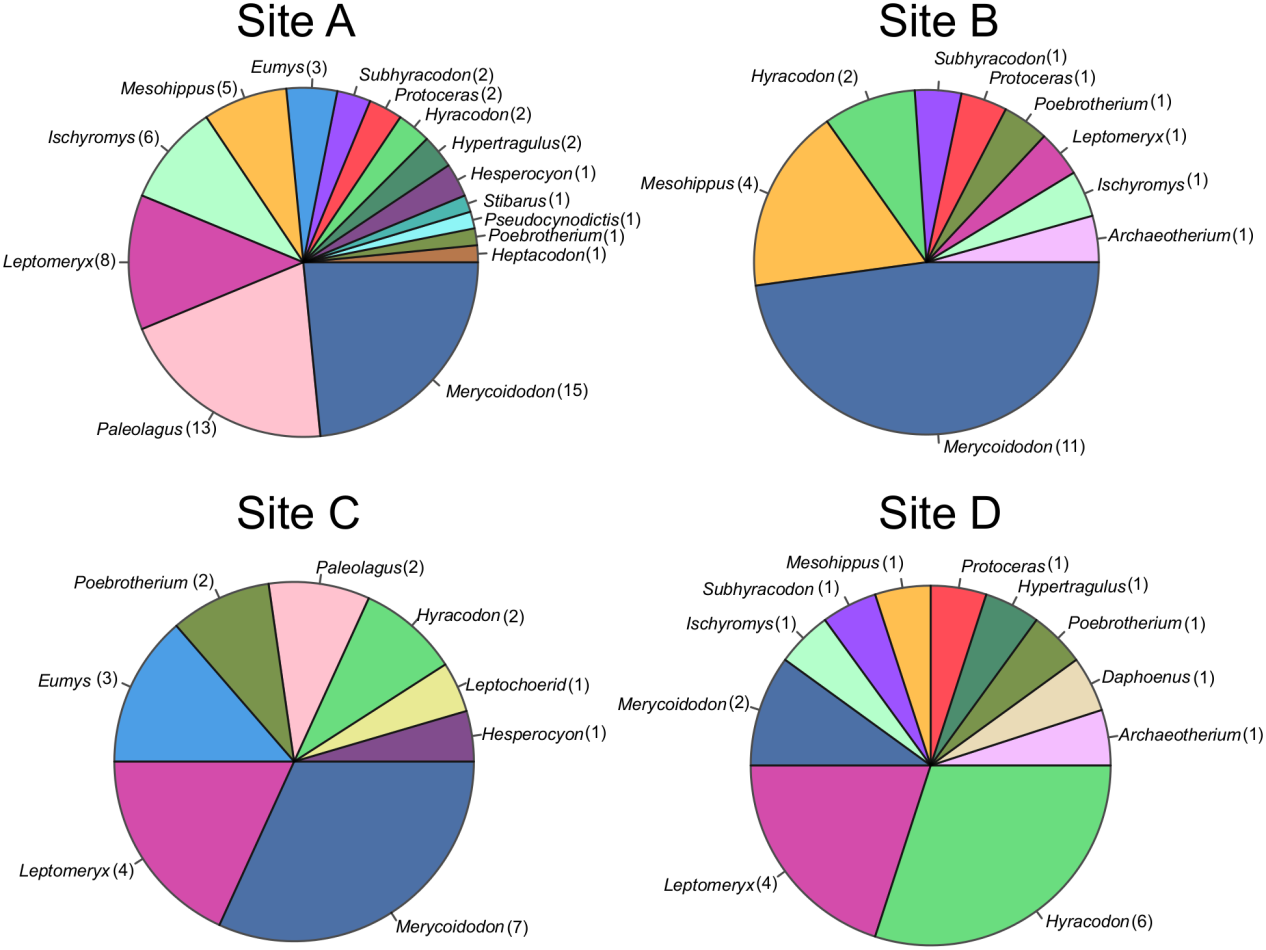
Distribution of Taxa by Facies. Distribution of taxa at sandstone versus siltstone hosted sites is indistinguishable in both environments. All specimens identifiable to genus are included. 87 specimens from the sandstone sites were included, 42 from the siltstone hosted sites.

**Fig 5:**
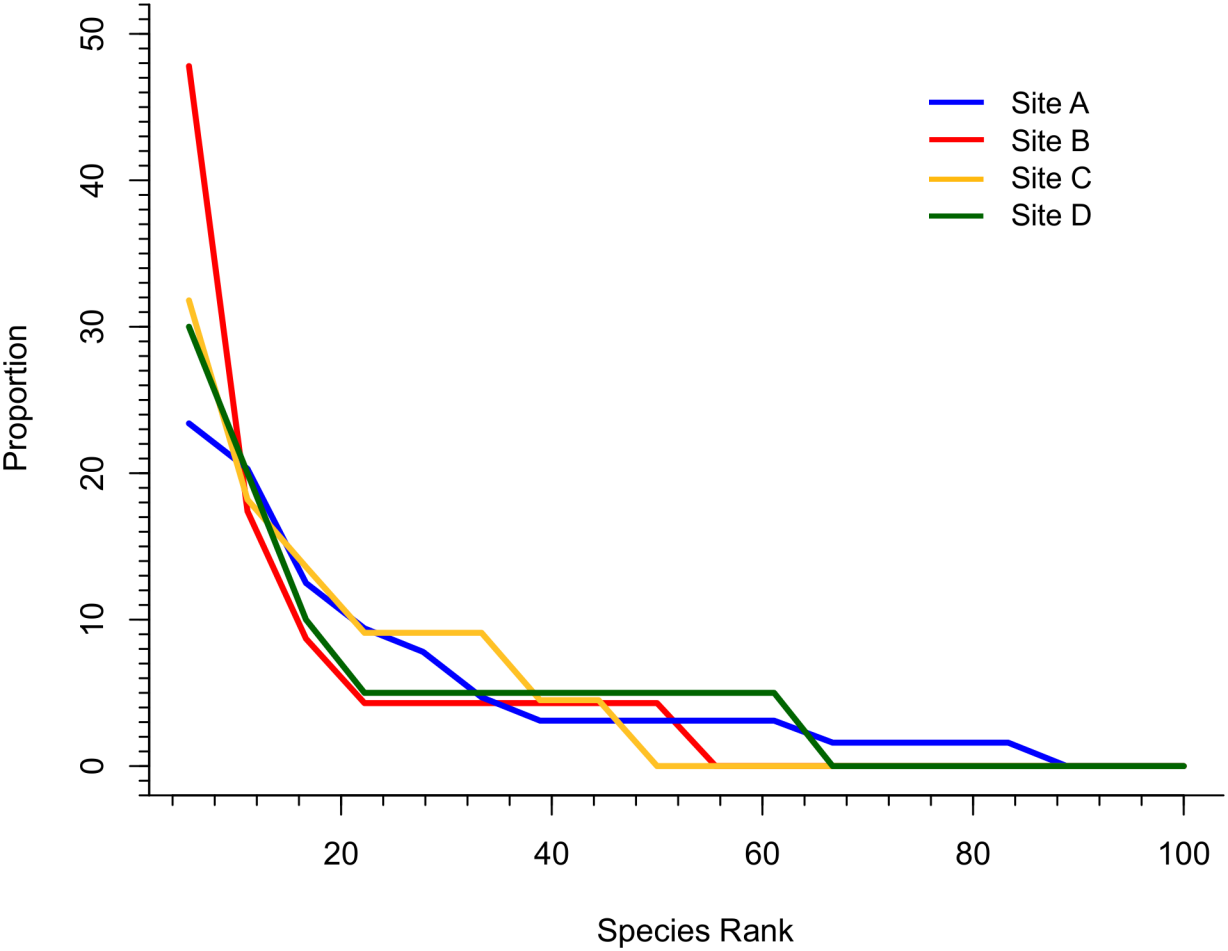
Rank Abundance Curves for Siltstone and Sandstone Assemblages. Proportion of specimens identified to a particular taxon plotted by abundance rank. The sites have been divided into sandstone and siltstone hosted groupings. Proportions given as percent of total number of specimens. Taxon ranked by proportion of the total number of taxa.

Examining the taxon abundance data (Fig 4), there is no statistically significant difference in taxon abundance distribution between siltstone-hosted and sandstone-hosted localities (*G*-test, *p* = 0.23), but it is worthy of note that sample sizes are sufficiently low that taxon abundances are likely very poorly constrained (observed percentage abundances are 95% likely to be within ~ ±15% of true abundances, using the method presented by Moore et al. [1]). Rank abundance curves are also similar between facies (Fig 5).

## Discussion

Of the four potential outcomes of the taphonomic and palaeoecological comparisons of the Poleslide Member assemblages (Table 2), our analyses demonstrate that, while the patterns of taphonomic modification exhibited by assemblages from different facies differ significantly, both diversity and taxon abundance structure are indistinguishable between facies. This has important implications for the interpretation of the taphonomic histories and palaeoecologies of the Poleslide assemblages, as it shows that taphofacies do exist in this unit, but means that ecological data can be meaningfully compared among assemblages collected from different facies.

### Taphonomic Histories

The taphonomic analyses undertaken in this study provide insight not only into the histories of each studied assemblage, but also regarding the taphonomic comparability of the Palmer Creek Poleslide assemblages and other similar assemblages. The main pattern observable in the taphonomic data is the differentiation of taphonomic modification between facies. We note that there is some intra-facies variation in taphonomic pattern (most notably in weathering stage distribution, and taxon size distribution), reflective of the complexities of terrestrial taphonomic histories. With only two samples from each facies, it is more difficult to identify the cause of such variation, and so interpretations of these taphonomic characteristic should be viewed with greater caution.

There is no difference in element size-frequency distribution between the two sampled facies, but the siltstone-hosted assemblages contain more elements from large taxa, are less weathered and abraded, and show a smaller proportion of tooth-bearing elements than those assemblages hosted in sandstones. It is apparent, therefore, that facies of preservation influences taphonomic history. This relationship has been noted numerous times for marine invertebrate assemblages [17,18,43], although a recent meta-analysis of terrestrial vertebrate fossil assemblages [27] found no relationship between pattern of taphonomic modification and facies. The latter study, however, included data that were aggregated from multiple localities within formations, and so is missing the fine-scale, facies-specific data that was collected as part of this study.

The overall taphonomic patterns of all of the Poleslide assemblages are similar to those from other intervals in the WRG quantified using the same methods as we present here [26], showing an overrepresentation of tooth-bearing elements, a generally low level of weathering and abrasion, and the importance of element density, surface area to volume ratio and element flatness in controlling the likelihood of element preservation. This gross similarity in taphonomic modification pattern over >2 million years indicates some constancy in the processes controlling the preservation of these faunas, independent of taxonomic composition and local sedimentological variation. Determining whether this long-term pattern of similarity is unique to the WRG or more broadly present in other terrestrial formations (which would indicate that regional-scale processes, for example patterns of accommodation fill, are the most important in controlling taphonomic modification) requires the application of similar analytical techniques to a broader range of vertebrate assemblages.

The fine-scale differences in taphonomic pattern between the Poleslide Member facies are more complex to explain. The increased degree of abrasion seen in the sandstone-hosted assemblages is not necessarily caused by the grain size of the encasing sediment. Experimental studies [44, 45] show that, in water, silt-sized sediment causes slightly more abrasion to large vertebrate bone fragments than coarse sand-sized sediment. Volcanic ashes and the loess derived from them (the primary component of the fine fraction of the Poleslide Member sediments) are the most abrasive sediment type [46]. Consequently the observed increased abrasion in sandstone-hosted assemblages cannot relate to the grain size or the composition of the encasing sediment. Instead, we hypothesize that the higher levels of abrasion observed in the sandstone-hosted elements are the result of a higher degree of sediment-bone interaction, although not necessarily a higher transport distance [47,48], due to more continuous, high velocity stream flow during the deposition of the sandstone facies, and the higher sediment carrying capacity of water. The sediment-bone interactions need not occur between the preserved bones and the encasing sediments–instead a significant proportion of the observed abrasion may be the action of suspended load of fine-grained sediments that are deposited further downstream. A further factor influencing degree of abrasion is the increased weathering shown by the sandstone-hosted elements. More weathered elements are markedly more easily abraded than elements that have not been exposed to significant weathering [44].

Why, then, do the sandstone-hosted assemblages show a higher degree of weathering than the siltstone-hosted assemblages? Bone weathering is controlled by a number of different factors including taxon and element, the microenvironment in which weathering has occurred, and the length of time for which the element has been exposed to weathering processes [49]. It should be noted that the mean increase in weathering stage observed in the Poleslide data is relatively small (0.13 units), and so it is not necessary to hypothesize a major difference in weathering history between facies. By averaging weathering stages across taxa and elements, we remove much of the influence of the first factors (given that the distributions of non-tooth-bearing elements are relatively similar across localities), and therefore the observed difference in weathering suggests that the sandstone hosted elements are exposed to a greater degree of weathering, are weathered for a longer period, or both. Given that the specimens forming these collections derived from a relatively large area, that each facies was represented by two samples from different stratigraphic horizons, and that, in the case of the sandstone-hosted assemblages, the specimens were likely not preserved in the same facies as they were weathered, it is less likely that weathering microenvironment plays a significant role in this difference. Therefore the sandstone-hosted assemblages must have been exposed to weathering processes for a slightly longer duration than the siltstone-hosted assemblages. While there is no direct evidence to suggest that elements preserved in channel deposits like those of the Poleslide are going to be buried below the taphonomically active zone more slowly than those in loessic deposits, the observed weathering pattern could be produced if elements preserved in the sandstone facies have experienced more episodes of reworking, and, consequently, recursive emergence into active weathering zones, than elements preserved in the siltstone facies.

The smaller proportion of tooth-bearing elements in the sandstone-hosted assemblages could be explained by a combination of two factors: the transport of the most easily transportable elements from the siltstone-hosted assemblages to the sandstone hosted assemblages (*i*.*e*., those elements that are small, low density and/or with significant amounts of buoyant fat present–generally not tooth-bearing elements–that can be moved by low energy flows), and the preferential destruction of less resilient non-tooth-bearing elements in overbank deposits. The relative influence of these two factors can be tested by comparing the distribution of other elements that are easy or difficult to transport between the two facies. This influence can also be tested by examining the proportion of bone fragments in each–a higher abundance of other more easily transportable elements in the fluvial assemblages would support transport of those elements from the siltstone facies to the sandstone facies, whereas a higher proportion of bone fragments in the aeolian assemblages would indicate that resilience to physical damage was the more important factor.

Our earlier taphonomic analyses show that sandstone facies assemblages have a higher proportion of more transportable elements, and the siltstone facies assemblages a higher proportion of less transportable elements. This implies some transport from the siltstone assemblages to the sandstone assemblages, and not vice versa. In turn, this would suggest that these overland flows must be low energy and likely not from major flooding, which would transfer material from streams to the floodplain. This is supported by the lack of fluvial sedimentary structures in the siltstone facies. The nature of sheet flow will place a limit on the size of element that can be transported, but further investigation of transport of fresh elements by such flows is necessary to understand these constraints, and hence the implications for the biasing of the Poleslide assemblages.

Bone fragments were not collected as part of this study, so the second factor cannot be directly tested. Instead, a rough proxy can be found in the proportion of non-dental elements collected that cannot be identified to generic level. As more fragmentary elements are (generally) more difficult to identify and as the preserved element proportions were relatively similar between non-dental elements, this index should approximate fragmentation. The siltstone-hosted assemblages show 71% unidentifiable specimens, based on this index, whereas the sandstone-hosted assemblages show 93%. These results indicate, albeit not conclusively, that transport of elements either from the siltstone-hosted assemblages or to the sandstone-hosted assemblages is responsible for this difference. Given the absence of sedimentary structures associated with water flow in the Palmer Creek Poleslide siltstones, this suggests that the siltstone-hosted assemblages will have less transport influence, so much of the difference in proportion of dental elements observed between facies is caused by the addition of more easily transportable material to the sandstone-hosted assemblages.

There is no significant difference in element size distribution between the two facies, indicating that the bias impacting Voorhies group distribution did not influence the distribution of larger or smaller elements. However, there was an increased abundance of larger size category taxa observed in the sandstone sites. While this pattern could be interpreted as a true ecological difference, the lack of observed diversity or compositional differences argues against such. Instead, we can suggest that smaller elements (and hence the remains of smaller taxa) are more likely to be destroyed in the Poleslide sandstones, hence decreasing the diversity of small taxa preserved there in comparison with the siltstone-hosted assemblages.

### Paleoecological Data

The similarity in mammalian diversity and composition of the Palmer Creek assemblages suggests that an ecological signal is recoverable despite differing patterns in taphonomic modification between the two respective facies. Larger sample sizes from each locality would be important to confirm this pattern, but assuming that these results are not artifacts of limited sample size, there are several important implications of these analyses. Firstly, this similarity implies that the paleoenvironmental preferences of the faunas preserved in both facies were similar. These assemblages are dominated by macro-mammals (>5 kg body mass), and it is unlikely that such taxa would ecologically segregate on the small length-scales of the Poleslide facies (most channel bodies are less than 30 m wide). While it is possible that the ecological homogeneity is caused by multiple rounds of reworking, and hence time averaging [50], there is little taphonomic and sedimentological evidence to indicate such large-scale reworking. As such, we suggest that the Palmer Creek faunas are preserving some aspects of the Oligocene paleofaunal structure of the Poleslide Member. These assemblages could, however, be subject to a systematic bias (for example the loss of all arboreal taxa) that would not be detected by the analyses herein. The most important use of these paleofaunal data is, therefore, to test for *changes* in ecological structure in a time series of similar assemblages, as systematic biases will not influence such studies. Such time series do not exist at present, but these results will facilitate their comparison.

## Conclusions

The presence of consistent ecological patterns between two facies showing different patterns of taphonomic modification in the Poleslide Member of the Brule Formation suggests that significant data regarding the taxonomic composition and abundance structure of ancient ecosystems can be recovered, even with a taphonomic overprint. Modern invertebrate assemblages have been demonstrated to preserve similar ecological data, despite varying taphonomic biases [51]. Should future analyses of this type (*i*.*e*., simultaneously addressing taphonomy and paleoecology) show that this pattern is more widespread in the vertebrate realm, and applicable across larger distances and more varied facies within a formation, the path would be opened for a much wider range of paleoecological comparisons among terrestrial vertebrate fossil assemblages. Assemblages collected throughout formations that preserve fossils in multiple facies could be combined to more accurately assess diversity and changes in ecosystem structure, greatly increasing our ability to study macro-scale ecological patterns in the vertebrate fossil record, and the results of existing studies conflating data from different facies to make ecological interpretations could be viewed with increased confidence.

The broad similarity of patterns of taphonomic modification between this study and others from the WRG suggests that the primary controls on such modification of terrestrial vertebrate fossil assemblages are large scale (regional/basinal), rather than local. Facies of preservation can, however, leave a final overprint on an assemblage. Further examination of such patterns on a larger scale will help identify the overarching controls on fossil preservation and modification, and so the degree to which vertebrate assemblages from different times and environments can be compared.

## Supporting Information

S1 Appendix R Code. Representative R code.
(DOCX)

S2 Appendix Full Dataset. Measured variables for all specimens studied in this analysis.
(XLSX)
