# Supplementary material for: Assessing the Control of Preservational Environment on Taphonomic and Ecological Patterns in an Oligocene Mammal Fauna from Badlands National Park, South Dakota
Source: PLoS One. 2016 Jun 15;11(6):e0157585. doi: 10.1371/journal.pone.0157585 (PMC4909216; doi:10.1371/journal.pone.0157585)
Supplement: S1 Appendix — Representative R code. (DOCX) [file pone.0157585.s001.docx]

Manova Analysis 2/22/13

> test.man.c.4to12<-manova(cbind(data.c[,4], data.c[,5], data.c[,6],data.c[,7],data.c[,8],data.c[,9],data.c[,10],data.c[,11],data.c[,12])~as.factor(data.c$Site))

> summary(test.man.c.4to12)

Df Pillai approx F num Df den Df Pr(>F)

as.factor(data.c$Site) 3 0.54472 12.227 27 1488 < 2.2e-16 ***

Residuals 502

---

Signif. codes: 0 ‘***’ 0.001 ‘**’ 0.01 ‘*’ 0.05 ‘.’ 0.1 ‘ ’ 1

> test.man.c.8to12<-manova(cbind(data.c[,8],data.c[,9],data.c[,10],data.c[,11],data.c[,12])~as.factor(data.c$Site))

> summary(test.man.c.8to12)

Df Pillai approx F num Df den Df Pr(>F)

as.factor(data.c$Site) 3 0.28794 13.993 15 1977 < 2.2e-16 ***

Residuals 661

---

Signif. codes: 0 ‘***’ 0.001 ‘**’ 0.01 ‘*’ 0.05 ‘.’ 0.1 ‘ ’ 1

> test.man.c.8to11<-manova(cbind(data.c[,8],data.c[,9],data.c[,10],data.c[,11])~as.factor(data.c$Site))

> summary(test.man.c.8to11)

Df Pillai approx F num Df den Df Pr(>F)

as.factor(data.c$Site) 3 0.18106 10.598 12 1980 < 2.2e-16 ***

Residuals 661

---

Signif. codes: 0 ‘***’ 0.001 ‘**’ 0.01 ‘*’ 0.05 ‘.’ 0.1 ‘ ’ 1

> data.lae<-read.csv("data.lae.csv")

> test.man.lae.4to12<-manova(cbind(data.lae[,4], data.lae[,5], data.lae[,6],data.lae[,7],data.lae[,8],data.lae[,9],data.lae[,10],data.lae[,11],data.lae[,12])~as.factor(data.lae$Site))

> test.man.lae.8to12<-manova(cbind(data.l[,8],data.lae[,9],data.lae[,10],data.lae[,11],data.lae[,12])~as.factor(data.lae$Site))

> test.man.lae.8to11<-manova(cbind(data.lae[,8],data.lae[,9],data.lae[,10],data.le[,11])~as.factor(data.l$Site))

> summary(test.man.4to12)

Error in summary(test.man.4to12) : object 'test.man.4to12' not found

> summary(test.man.lae.4to12)

Df Pillai approx F num Df den Df Pr(>F)

as.factor(data.l$Site) 1 0.23661 10.125 9 294 1.411e-13 ***

Residuals 302

---

Signif. codes: 0 ‘***’ 0.001 ‘**’ 0.01 ‘*’ 0.05 ‘.’ 0.1 ‘ ’ 1

> summary(test.man.lae.8to12)

Df Pillai approx F num Df den Df Pr(>F)

as.factor(data.l$Site) 1 0.064074 4.1898 5 306 0.001069 **

Residuals 310

---

Signif. codes: 0 ‘***’ 0.001 ‘**’ 0.01 ‘*’ 0.05 ‘.’ 0.1 ‘ ’ 1

> summary(test.man.lae.8to11)

Df Pillai approx F num Df den Df Pr(>F)

as.factor(data.l$Site) 1 0.019905 1.5587 4 307 0.1852

Residuals 310

> data.cae<-read.csv("data.c.ae.csv")

> test.man.cae.4to12<-manova(cbind(data.cae[,4], data.cae[,5], data.cae[,6],data.cae[,7],data.cae[,8],data.cae[,9],data.cae[,10],data.cae[,11],data.cae[,12])~as.factor(data.cae$Site))

> test.man.cae.8to12<-manova(cbind(data.cae[,8],data.cae[,9],data.cae[,10],data.cae[,11],data.cae[,12])~as.factor(data.cae$Site))

> test.man.cae.8to11<-manova(cbind(data.cae[,8],data.cae[,9],data.cae[,10],data.cae[,11])~as.factor(data.cae$Site))

> summary(test.man.cae.4to12)

Df Pillai approx F num Df den Df Pr(>F)

as.factor(data.cae$Site) 1 0.23856 10.269 9 295 8.792e-14 ***

Residuals 303

---

Signif. codes: 0 ‘***’ 0.001 ‘**’ 0.01 ‘*’ 0.05 ‘.’ 0.1 ‘ ’ 1

> summary(test.man.cae.8to12)

Df Pillai approx F num Df den Df Pr(>F)

as.factor(data.cae$Site) 1 0.063875 4.1895 5 307 0.001068 **

Residuals 311

---

Signif. codes: 0 ‘***’ 0.001 ‘**’ 0.01 ‘*’ 0.05 ‘.’ 0.1 ‘ ’ 1

> summary(test.man.cae.8to11)

Df Pillai approx F num Df den Df Pr(>F)

as.factor(data.cae$Site) 1 0.020196 1.5872 4 308 0.1775

Residuals 311

> data.lfl<-read.csv("data.l.fl.csv")

> test.man.lfl.4to12<-manova(cbind(data.lfl[,4], data.lfl[,5], data.lfl[,6],data.lfl[,7],data.lfl[,8],data.lfl[,9],data.lfl[,10],data.lfl[,11],data.lfl[,12])~as.factor(data.lfl$Site))

> test.man.lfl.8to12<-manova(cbind(data.lfl[,8],data.lfl[,9],data.lfl[,10],data.lfl[,11],data.lfl[,12])~as.factor(data.lfl$Site))

> test.man.lfl.8to11<-manova(cbind(data.lfl[,8],data.lfl[,9],data.lfl[,10],data.lfl[,11])~as.factor(data.lfl$Site))

> summary(test.man.lfl.4to12)

Df Pillai approx F num Df den Df Pr(>F)

as.factor(data.lfl$Site) 1 0.24124 6.7475 9 191 2.181e-08 ***

Residuals 199

---

Signif. codes: 0 ‘***’ 0.001 ‘**’ 0.01 ‘*’ 0.05 ‘.’ 0.1 ‘ ’ 1

> summary(test.man.lfl.8to12)

Df Pillai approx F num Df den Df Pr(>F)

as.factor(data.lfl$Site) 1 0.11914 9.387 5 347 2.101e-08 ***

Residuals 351

---

Signif. codes: 0 ‘***’ 0.001 ‘**’ 0.01 ‘*’ 0.05 ‘.’ 0.1 ‘ ’ 1

> summary(test.man.lfl.8to11)

Df Pillai approx F num Df den Df Pr(>F)

as.factor(data.lfl$Site) 1 0.047012 4.2918 4 348 0.002109 **

Residuals 351

---

Signif. codes: 0 ‘***’ 0.001 ‘**’ 0.01 ‘*’ 0.05 ‘.’ 0.1 ‘ ’ 1

> data.cfl<-read.csv("data.c.fl.csv")

> test.man.cfl.4to12<-manova(cbind(data.cfl[,4], data.cfl[,5], data.cfl[,6],data.cfl[,7],data.cfl[,8],data.cfl[,9],data.cfl[,10],data.cfl[,11],data.cfl[,12])~as.factor(data.cfl$Site))

> test.man.cfl.8to12<-manova(cbind(data.cfl[,8],data.cfl[,9],data.cfl[,10],data.cfl[,11],data.cfl[,12])~as.factor(data.cfl$Site))

> test.man.cfl.8to11<-manova(cbind(data.cfl[,8],data.cfl[,9],data.cfl[,10],data.cfl[,11])~as.factor(data.cfl$Site))

> summary(test.man.cfl.4to12)

Df Pillai approx F num Df den Df Pr(>F)

as.factor(data.cfl$Site) 1 0.23561 6.5412 9 191 4.087e-08 ***

Residuals 199

---

Signif. codes: 0 ‘***’ 0.001 ‘**’ 0.01 ‘*’ 0.05 ‘.’ 0.1 ‘ ’ 1

> summary(test.man.cfl.8to12)

Df Pillai approx F num Df den Df Pr(>F)

as.factor(data.cfl$Site) 1 0.11392 8.8969 5 346 5.815e-08 ***

Residuals 350

---

Signif. codes: 0 ‘***’ 0.001 ‘**’ 0.01 ‘*’ 0.05 ‘.’ 0.1 ‘ ’ 1

> summary(test.man.cfl.8to11)

Df Pillai approx F num Df den Df Pr(>F)

as.factor(data.cfl$Site) 1 0.043875 3.9808 4 347 0.003584 **

Residuals 350

---

Signif. codes: 0 ‘***’ 0.001 ‘**’ 0.01 ‘*’ 0.05 ‘.’ 0.1 ‘ ’ 1

> data.l<-read.csv("DataL.csv")

> test.man.l.4to12<-manova(cbind(data.l[,4], data.l[,5], data.l[,6],data.l[,7],data.l[,8],data.l[,9],data.l[,10],data.l[,11],data.l[,12])~as.factor(data.l$Site))

> test.man.l.8to12<-manova(cbind(data.l[,8],data.l[,9],data.l[,10],data.l[,11],data.l[,12])~as.factor(data.l$Site))

> test.man.l.8to11<-manova(cbind(data.l[,8],data.l[,9],data.l[,10],data.l[,11])~as.factor(data.l$Site))

> summary(test.man.4to12)

Error in summary(test.man.4to12) : object 'test.man.4to12' not found

> summary(test.man.l.4to12)

Df Pillai approx F num Df den Df Pr(>F)

as.factor(data.l$Site) 3 0.55057 12.363 27 1485 < 2.2e-16 ***

Residuals 501

---

Signif. codes: 0 ‘***’ 0.001 ‘**’ 0.01 ‘*’ 0.05 ‘.’ 0.1 ‘ ’ 1

> summary(test.man.l.8to12)

Df Pillai approx F num Df den Df Pr(>F)

as.factor(data.l$Site) 3 0.29115 14.166 15 1977 < 2.2e-16 ***

Residuals 661

---

Signif. codes: 0 ‘***’ 0.001 ‘**’ 0.01 ‘*’ 0.05 ‘.’ 0.1 ‘ ’ 1

> summary(test.man.l.8to11)

Df Pillai approx F num Df den Df Pr(>F)

as.factor(data.l$Site) 3 0.18537 10.867 12 1980 < 2.2e-16 ***

Residuals 661

---

Signif. codes: 0 ‘***’ 0.001 ‘**’ 0.01 ‘*’ 0.05 ‘.’ 0.1 ‘ ’ 1

> test.man.laf.4to12<-manova(cbind(data.l[,4], data.l[,5], data.l[,6],data.l[,7],data.l[,8],data.l[,9],data.l[,10],data.l[,11],data.l[,12])~as.factor(data.l$AF))

> test.man.laf.8to12<-manova(cbind(data.l[,8],data.l[,9],data.l[,10],data.l[,11],data.l[,12])~as.factor(data.l$AF))

> test.man.laf.8to11<-manova(cbind(data.l[,8],data.l[,9],data.l[,10],data.l[,11])~as.factor(data.l$AF))

> summary(test.man.laf.4to12)

Df Pillai approx F num Df den Df Pr(>F)

as.factor(data.l$AF) 1 0.25272 18.6 9 495 < 2.2e-16 ***

Residuals 503

---

Signif. codes: 0 ‘***’ 0.001 ‘**’ 0.01 ‘*’ 0.05 ‘.’ 0.1 ‘ ’ 1

> summary(test.man.laf.8to12)

Df Pillai approx F num Df den Df Pr(>F)

as.factor(data.l$AF) 1 0.23617 40.751 5 659 < 2.2e-16 ***

Residuals 663

---

Signif. codes: 0 ‘***’ 0.001 ‘**’ 0.01 ‘*’ 0.05 ‘.’ 0.1 ‘ ’ 1

> summary(test.man.laf.8to11)

Df Pillai approx F num Df den Df Pr(>F)

as.factor(data.l$AF) 1 0.15091 29.325 4 660 < 2.2e-16 ***

Residuals 663

---

Signif. codes: 0 ‘***’ 0.001 ‘**’ 0.01 ‘*’ 0.05 ‘.’ 0.1 ‘ ’ 1

> data.c<-read.csv("DataC.csv")

> test.man.caf.4to12<-manova(cbind(data.c[,4], data.c[,5], data.c[,6],data.c[,7],data.c[,8],data.c[,9],data.c[,10],data.c[,11],data.c[,12])~as.factor(data.c$AF))

> test.man.caf.8to12<-manova(cbind(data.c[,8],data.c[,9],data.c[,10],data.c[,11],data.c[,12])~as.factor(data.c$AF))

> test.man.caf.8to11<-manova(cbind(data.c[,8],data.c[,9],data.c[,10],data.c[,11])~as.factor(data.c$AF))

> summary(test.man.4to12)

Error in summary(test.man.4to12) : object 'test.man.4to12' not found

> summary(test.man.caf.4to12)

Df Pillai approx F num Df den Df Pr(>F)

as.factor(data.c$AF) 1 0.24822 18.197 9 496 < 2.2e-16 ***

Residuals 504

---

Signif. codes: 0 ‘***’ 0.001 ‘**’ 0.01 ‘*’ 0.05 ‘.’ 0.1 ‘ ’ 1

> summary(test.man.caf.8to12)

Df Pillai approx F num Df den Df Pr(>F)

as.factor(data.c$AF) 1 0.23477 40.435 5 659 < 2.2e-16 ***

Residuals 663

---

Signif. codes: 0 ‘***’ 0.001 ‘**’ 0.01 ‘*’ 0.05 ‘.’ 0.1 ‘ ’ 1

> summary(test.man.caf.8to11)

Df Pillai approx F num Df den Df Pr(>F)

as.factor(data.c$AF) 1 0.14817 28.7 4 660 < 2.2e-16 ***

Residuals 663

---

Signif. codes: 0 ‘***’ 0.001 ‘**’ 0.01 ‘*’ 0.05 ‘.’ 0.1 ‘ ’ 1

> test.man.LDAB<-manova(cbind(L.DAB[,4], L.DAB[,5], L.DAB[,6], L.DAB[,7], L.DAB[,8], L.DAB[,9], L.DAB[,10], L.DAB[,11], L.DAB[,12])~as.factor(L.DAB$DAB))

> summary(test.man.LDAB)

Df Pillai approx F num Df den Df Pr(>F)

as.factor(L.DAB$DAB) 1 0.27208 19.354 9 466 < 2.2e-16 ***

Residuals 474

---

Signif. codes: 0 ‘***’ 0.001 ‘**’ 0.01 ‘*’ 0.05 ‘.’ 0.1 ‘ ’ 1

> test.man.LDAB<-manova(cbind(L.DAB[,8], L.DAB[,9], L.DAB[,10], L.DAB[,11], L.DAB[,12])~as.factor(L.DAB$DAB))

> summary(test.man.LDAB)

Df Pillai approx F num Df den Df Pr(>F)

as.factor(L.DAB$DAB) 1 0.1849 22.094 5 487 < 2.2e-16 ***

Residuals 491

---

Signif. codes: 0 ‘***’ 0.001 ‘**’ 0.01 ‘*’ 0.05 ‘.’ 0.1 ‘ ’ 1

> test.man.LDAB<-manova(cbind(L.DAB[,8], L.DAB[,9], L.DAB[,10], L.DAB[,11])~as.factor(L.DAB$DAB))

> summary(test.man.LDAB)

Df Pillai approx F num Df den Df Pr(>F)

as.factor(L.DAB$DAB) 1 0.14645 20.932 4 488 6.095e-16 ***

Residuals 491

---

Signif. codes: 0 ‘***’ 0.001 ‘**’ 0.01 ‘*’ 0.05 ‘.’ 0.1 ‘ ’ 1

> test.man.CDAB<-manova(cbind(C.DAB[,4], C.DAB[,5], C.DAB[,6], C.DAB[,7], C.DAB[,8], C.DAB[,9], C.DAB[,10], C.DAB[,11], C.DAB[,12])~as.factor(C.DAB$DAB))

> summary(test.man.CDAB)

Df Pillai approx F num Df den Df Pr(>F)

as.factor(C.DAB$DAB) 1 0.26407 18.619 9 467 < 2.2e-16 ***

Residuals 475

---

Signif. codes: 0 ‘***’ 0.001 ‘**’ 0.01 ‘*’ 0.05 ‘.’ 0.1 ‘ ’ 1

> test.man.CDAB<-manova(cbind(C.DAB[,8], C.DAB[,9], C.DAB[,10], C.DAB[,11], C.DAB[,12])~as.factor(C.DAB$DAB))

> summary(test.man.CDAB)

Df Pillai approx F num Df den Df Pr(>F)

as.factor(C.DAB$DAB) 1 0.1818 21.642 5 487 < 2.2e-16 ***

Residuals 491

---

Signif. codes: 0 ‘***’ 0.001 ‘**’ 0.01 ‘*’ 0.05 ‘.’ 0.1 ‘ ’ 1

> test.man.CDAB<-manova(cbind(C.DAB[,8], C.DAB[,9], C.DAB[,10], C.DAB[,11])~as.factor(C.DAB$DAB))

> summary(test.man.CDAB)

Df Pillai approx F num Df den Df Pr(>F)

as.factor(C.DAB$DAB) 1 0.14076 19.986 4 488 2.964e-15 ***

Residuals 491

---

Signif. codes: 0 ‘***’ 0.001 ‘**’ 0.01 ‘*’ 0.05 ‘.’ 0.1 ‘ ’ 1

> test.man.LCAB<-manova(cbind(L.CAB[,4], L.CAB[,5], L.CAB[,6], L.CAB[,7], L.CAB[,8], L.CAB[,9], L.CAB[,10], L.CAB[,11], L.CAB[,12])~as.factor(L.CAB$CAB))

> summary(test.man.LCAB)

Df Pillai approx F num Df den Df Pr(>F)

as.factor(L.CAB$CAB) 1 0.28004 13.959 9 323 < 2.2e-16 ***

Residuals 331

---

Signif. codes: 0 ‘***’ 0.001 ‘**’ 0.01 ‘*’ 0.05 ‘.’ 0.1 ‘ ’ 1

> test.man.LCAB<-manova(cbind(L.CAB[,8], L.CAB[,9], L.CAB[,10], L.CAB[,11], L.CAB[,12])~as.factor(L.CAB$CAB))

> summary(test.man.LCAB)

Df Pillai approx F num Df den Df Pr(>F)

as.factor(L.CAB$CAB) 1 0.18984 22.401 5 478 < 2.2e-16 ***

Residuals 482

---

Signif. codes: 0 ‘***’ 0.001 ‘**’ 0.01 ‘*’ 0.05 ‘.’ 0.1 ‘ ’ 1

> test.man.LCAB<-manova(cbind(L.CAB[,8], L.CAB[,9], L.CAB[,10], L.CAB[,11])~as.factor(L.CAB$CAB))

> summary(test.man.LCAB)

Df Pillai approx F num Df den Df Pr(>F)

as.factor(L.CAB$CAB) 1 0.13674 18.969 4 479 1.713e-14 ***

Residuals 482

---

Signif. codes: 0 ‘***’ 0.001 ‘**’ 0.01 ‘*’ 0.05 ‘.’ 0.1 ‘ ’ 1

> test.man.CCAB<-manova(cbind(C.CAB[,4], C.CAB[,5], C.CAB[,6], C.CAB[,7], C.CAB[,8], C.CAB[,9], C.CAB[,10], C.CAB[,11], C.CAB[,12])~as.factor(C.CAB$CAB))

> summary(test.man.CCAB)

Df Pillai approx F num Df den Df Pr(>F)

as.factor(C.CAB$CAB) 1 0.28013 14.009 9 324 < 2.2e-16 ***

Residuals 332

---

Signif. codes: 0 ‘***’ 0.001 ‘**’ 0.01 ‘*’ 0.05 ‘.’ 0.1 ‘ ’ 1

> test.man.CCAB<-manova(cbind(C.CAB[,8], C.CAB[,9], C.CAB[,10], C.CAB[,11], C.CAB[,12])~as.factor(C.CAB$CAB))

> summary(test.man.CCAB)

Df Pillai approx F num Df den Df Pr(>F)

as.factor(C.CAB$CAB) 1 0.1884 22.239 5 479 < 2.2e-16 ***

Residuals 483

---

Signif. codes: 0 ‘***’ 0.001 ‘**’ 0.01 ‘*’ 0.05 ‘.’ 0.1 ‘ ’ 1

> test.man.CCAB<-manova(cbind(C.CAB[,8], C.CAB[,9], C.CAB[,10], C.CAB[,11])~as.factor(C.CAB$CAB))

> summary(test.man.CCAB)

Df Pillai approx F num Df den Df Pr(>F)

as.factor(C.CAB$CAB) 1 0.1353 18.776 4 480 2.36e-14 ***

Residuals 483

---

Signif. codes: 0 ‘***’ 0.001 ‘**’ 0.01 ‘*’ 0

Summary NMDS Analysis 10/3/15

> library(vegan)

Loading required package: permute

This is vegan 2.0-3

> X412<-read.csv("NMDS 412.csv")

Warning message:

In read.table(file = file, header = header, sep = sep, quote = quote, :

incomplete final line found by readTableHeader on 'NMDS 412.csv'

> summary.nmds<-metaMDS(comm=decostand(X412[,c(2:10)], "max", distance="euclidean", k=2, trymax=25, autotransform=F, zerodist="add"))

Run 0 stress 0

Run 1 stress 0

... procrustes: rmse 0.1085404 max resid 0.1635072

Run 2 stress 0

... procrustes: rmse 0.1095997 max resid 0.1215021

Run 3 stress 0

... procrustes: rmse 0.01444225 max resid 0.01831314

Run 4 stress 0

... procrustes: rmse 0.1838846 max resid 0.279758

Run 5 stress 0

... procrustes: rmse 0.07610383 max resid 0.09153763

Run 6 stress 0.0001010799

... procrustes: rmse 0.2135848 max resid 0.3304898

Run 7 stress 0

... procrustes: rmse 0.0504003 max resid 0.07270056

Run 8 stress 0

... procrustes: rmse 0.06059457 max resid 0.07508873

Run 9 stress 9.805452e-05

... procrustes: rmse 0.2135805 max resid 0.3304829

Run 10 stress 0

... procrustes: rmse 0.1633826 max resid 0.2159157

Run 11 stress 0

... procrustes: rmse 0.06865776 max resid 0.08570652

Run 12 stress 0

... procrustes: rmse 0.1862472 max resid 0.283301

Run 13 stress 0

... procrustes: rmse 0.03353399 max resid 0.04626285

Run 14 stress 8.968219e-05

... procrustes: rmse 0.2135648 max resid 0.3304584

Run 15 stress 0

... procrustes: rmse 0.08181731 max resid 0.1262139

Run 16 stress 0

... procrustes: rmse 0.1596117 max resid 0.2362362

Run 17 stress 0

... procrustes: rmse 0.05730045 max resid 0.07685456

Run 18 stress 7.9568e-05

... procrustes: rmse 0.2135534 max resid 0.3304401

Run 19 stress 0

... procrustes: rmse 0.08886095 max resid 0.1128138

Run 20 stress 0

... procrustes: rmse 0.1146469 max resid 0.1697302

Warning message:

In postMDS(out$points, dis, plot = max(0, plot - 1), ...) :

skipping half-change scaling: too few points below threshold

> plot(summary.nmds)

> orditorp(summary.nmds, display="sites", label=X412$Site)

> test<-orditorp(summary.nmds)

Error in match.arg(display) :

argument "display" is missing, with no default

> identify(test, "site", labels=X412$Site)

Error in identify(test, "site", labels = X412$Site) :

object 'test' not found

> test<-ordiplot(summary.nmds)

> identify(test, "site", labels=X412$Site)

[1] 1 2 3 4

> identify(test, "species")

[1] 1 2 3 4 5 6 7 8 9

> NMDS411<-metaMDS(comm=decostand(X412[,c(2:9)], "max", distance="euclidean", k=2, trymax=25, autotransform=F, zerodist="add"))

Run 0 stress 0

Run 1 stress 0

... procrustes: rmse 0.1293418 max resid 0.1902209

Run 2 stress 0

... procrustes: rmse 0.1427446 max resid 0.229821

Run 3 stress 0

... procrustes: rmse 0.118208 max resid 0.1741433

Run 4 stress 0

... procrustes: rmse 0.06936806 max resid 0.0955439

Run 5 stress 0

... procrustes: rmse 0.07477095 max resid 0.1105151

Run 6 stress 7.919542e-05

... procrustes: rmse 0.3141216 max resid 0.5051751

Run 7 stress 0

... procrustes: rmse 0.1243451 max resid 0.1913419

Run 8 stress 0

... procrustes: rmse 0.08854755 max resid 0.1311

Run 9 stress 0

... procrustes: rmse 0.2687101 max resid 0.4208058

Run 10 stress 0

... procrustes: rmse 0.226824 max resid 0.3192164

Run 11 stress 0

... procrustes: rmse 0.2450604 max resid 0.4088679

Run 12 stress 0

... procrustes: rmse 0.1998342 max resid 0.335897

Run 13 stress 0

... procrustes: rmse 0.08098782 max resid 0.1087136

Run 14 stress 0

... procrustes: rmse 0.2787144 max resid 0.4074262

Run 15 stress 0

... procrustes: rmse 0.1665217 max resid 0.2409898

Run 16 stress 0

... procrustes: rmse 0.1102779 max resid 0.1503919

Run 17 stress 7.79273e-05

... procrustes: rmse 0.314138 max resid 0.5051682

Run 18 stress 0

... procrustes: rmse 0.1971992 max resid 0.3136137

Run 19 stress 0

... procrustes: rmse 0.1804993 max resid 0.3038753

Run 20 stress 0

... procrustes: rmse 0.1817669 max resid 0.2751719

Warning message:

In postMDS(out$points, dis, plot = max(0, plot - 1), ...) :

skipping half-change scaling: too few points below threshold

> plot(NMDS411)

> orditorp(NMDS411, display="sites", label=X412$Site)

> test<-ordiplot(NMDS411)

> identify(test, "site", labels=X412$Site)

[1] 1 2 3 4

> identify(test, "species")

[1] 1 2 3 4 5 6 7 8

> NMDS812<-metaMDS(comm=decostand(X412[,c(6:10)], "max", distance="euclidean", k=2, trymax=25, autotransform=F, zerodist="add"))

Run 0 stress 0

Run 1 stress 0

... procrustes: rmse 0.1904487 max resid 0.2539937

Run 2 stress 0

... procrustes: rmse 0.2106224 max resid 0.2616179

Run 3 stress 0

... procrustes: rmse 0.1809984 max resid 0.2369793

Run 4 stress 0

... procrustes: rmse 0.1423114 max resid 0.1791503

Run 5 stress 0

... procrustes: rmse 0.2209785 max resid 0.3380915

Run 6 stress 0

... procrustes: rmse 0.07914779 max resid 0.1149303

Run 7 stress 0

... procrustes: rmse 0.2042473 max resid 0.2936519

Run 8 stress 0

... procrustes: rmse 0.1367697 max resid 0.1866046

Run 9 stress 0

... procrustes: rmse 0.2368661 max resid 0.3411093

Run 10 stress 0

... procrustes: rmse 0.1438483 max resid 0.1803769

Run 11 stress 0

... procrustes: rmse 0.1063653 max resid 0.1597606

Run 12 stress 0.169102

Run 13 stress 0

... procrustes: rmse 0.2323525 max resid 0.274294

Run 14 stress 0

... procrustes: rmse 0.2013859 max resid 0.2622937

Run 15 stress 0.169102

Run 16 stress 0

... procrustes: rmse 0.189724 max resid 0.2387449

Run 17 stress 0

... procrustes: rmse 0.1981962 max resid 0.2345564

Run 18 stress 0

... procrustes: rmse 0.1895056 max resid 0.2283388

Run 19 stress 0

... procrustes: rmse 0.2002296 max resid 0.2362104

Run 20 stress 0

... procrustes: rmse 0.2039241 max resid 0.2511233

Warning message:

In postMDS(out$points, dis, plot = max(0, plot - 1), ...) :

skipping half-change scaling: too few points below threshold

> plot(NMDS812)

> orditorp(NMDS812, display="sites", label=X412$Site)

> test<-ordiplot(NMDS812)

> identify(test, "site", labels=X412$Site)

[1] 1 2 3 4

> identify(test, "species")

[1] 1 2 3 4 5

> NMDS811<-metaMDS(comm=decostand(X412[,c(6:9)], "max", distance="euclidean", k=2, trymax=25, autotransform=F, zerodist="add"))

Run 0 stress 0

Run 1 stress 0

... procrustes: rmse 0.1841578 max resid 0.250006

Run 2 stress 0

... procrustes: rmse 0.1379473 max resid 0.1638341

Run 3 stress 0

... procrustes: rmse 0.2156207 max resid 0.2773663

Run 4 stress 0

... procrustes: rmse 0.2855641 max resid 0.3815948

Run 5 stress 0

... procrustes: rmse 0.2010115 max resid 0.2684392

Run 6 stress 0

... procrustes: rmse 0.1339864 max resid 0.1697039

Run 7 stress 0

... procrustes: rmse 0.08231897 max resid 0.1182697

Run 8 stress 0

... procrustes: rmse 0.1360212 max resid 0.1842224

Run 9 stress 0

... procrustes: rmse 0.1995341 max resid 0.2373581

Run 10 stress 0

... procrustes: rmse 0.2095442 max resid 0.2902123

Run 11 stress 0

... procrustes: rmse 0.2242838 max resid 0.2889385

Run 12 stress 0

... procrustes: rmse 0.2371374 max resid 0.3068632

Run 13 stress 0

... procrustes: rmse 0.1442965 max resid 0.1728721

Run 14 stress 0

... procrustes: rmse 0.1370074 max resid 0.1800094

Run 15 stress 0

... procrustes: rmse 0.1245357 max resid 0.1382485

Run 16 stress 0

... procrustes: rmse 0.158706 max resid 0.1885701

Run 17 stress 0

... procrustes: rmse 0.2414393 max resid 0.3135388

Run 18 stress 0

... procrustes: rmse 0.1129803 max resid 0.1574385

Run 19 stress 0

... procrustes: rmse 0.2179399 max resid 0.2874462

Run 20 stress 0

... procrustes: rmse 0.1102608 max resid 0.1334947

Warning message:

In postMDS(out$points, dis, plot = max(0, plot - 1), ...) :

skipping half-change scaling: too few points below threshold

> plot(NMDS811)

> orditorp(NMDS811, display="sites", label=X412$Site)

> test<-ordiplot(NMDS811)

> identify(test, "site", labels=X412$Site)

[1] 1 2 3 4

> identify(test, "species")

[1] 1 2 3 4

Initial NMDS Analysis of Species Distribution 3/9/14

> Elements_ABCD[,c(2:21)]

Astragalus Calcaneum Cranium Dentary Femur Fibula Humerus Innominate

Site A 6 6 3 0 6 0 6 4

Site B 4 0 6 1 15 0 4 1

Site C 2 9 7 1 5 1 6 3

Site D 11 4 3 0 19 0 12 5

Maxilla Metapodial Phalanx Podial Radius Rib Scapula Sesamoid Tibia

Site A 0 14 7 2 2 1 5 0 11

Site B 0 14 6 2 2 0 1 0 8

Site C 0 21 30 16 1 3 2 1 8

Site D 1 12 27 10 3 1 9 2 17

Tooth.Bearing.Elements Ulna Vertebra

Site A 77 1 28

Site B 45 1 24

Site C 15 2 39

Site D 16 3 27

> Elements_All.NMDS<-metaMDS(comm=decostand(Elements_ABCD[,c(2:21)], "max", distance="bray", k=2, trymax=25, autotransform=F, zerodist="add"))

Run 0 stress 0

Run 1 stress 0

... procrustes: rmse 0.0535778 max resid 0.06450759

Run 2 stress 7.773251e-05

... procrustes: rmse 0.193207 max resid 0.2526644

Run 3 stress 5.905348e-05

... procrustes: rmse 0.1612304 max resid 0.2339706

Run 4 stress 0.169102

Run 5 stress 0

... procrustes: rmse 0.0573616 max resid 0.06496104

Run 6 stress 0

... procrustes: rmse 0.1305119 max resid 0.1910113

Run 7 stress 0

... procrustes: rmse 0.1157404 max resid 0.1587055

Run 8 stress 0

... procrustes: rmse 0.1575029 max resid 0.2266023

Run 9 stress 0

... procrustes: rmse 0.05541791 max resid 0.07399821

Run 10 stress 0

... procrustes: rmse 0.04780773 max resid 0.05727568

Run 11 stress 0

... procrustes: rmse 0.149721 max resid 0.2189968

Run 12 stress 0

... procrustes: rmse 0.1903653 max resid 0.2436338

Run 13 stress 0.169102

Run 14 stress 0

... procrustes: rmse 0.09539533 max resid 0.1261168

Run 15 stress 0

... procrustes: rmse 0.1371032 max resid 0.1951691

Run 16 stress 0

... procrustes: rmse 0.1603634 max resid 0.1837666

Run 17 stress 0.169102

Run 18 stress 0

... procrustes: rmse 0.1338366 max resid 0.1678922

Run 19 stress 0.169102

Run 20 stress 0

... procrustes: rmse 0.08382924 max resid 0.0939028

Warning message:

In postMDS(out$points, dis, plot = max(0, plot - 1), ...) :

skipping half-change scaling: too few points below threshold

> Elements_All.NMDS.Plot<-ordiplot(Elements_All.NMDS)

> identify(Elements_All.NMDS.Plot, "sites", labels=Elements_ABCD$Site)

[1] 1 2 3 4

GREATER THAN OR EQUAL TO 5

> Elements_ABCD[,c(2,3,4,6,8,9,11,12,13,14,16,18,19,20,21)]

Astragalus Calcaneum Cranium Femur Humerus Innominate Metapodial Phalanx

Site A 6 6 3 6 6 4 14 7

Site B 4 0 6 15 4 1 14 6

Site C 2 9 7 5 6 3 21 30

Site D 11 4 3 19 12 5 12 27

Podial Radius Scapula Tibia Tooth.Bearing.Elements Ulna Vertebra

Site A 2 2 5 11 77 1 28

Site B 2 2 1 8 45 1 24

Site C 16 1 2 8 15 2 39

Site D 10 3 9 17 16 3 27

> Elements_5.NMDS<-metaMDS(comm=decostand(Elements_ABCD[,c(2,3,4,6,8,9,11,12,13,14,16,18,19,20,21)], "max", distance="bray", k=2, trymax=25, autotransform=F, zerodist="add"))

Run 0 stress 0

Run 1 stress 0

... procrustes: rmse 0.05346077 max resid 0.08249621

Run 2 stress 0

... procrustes: rmse 0.05149973 max resid 0.08551588

Run 3 stress 0

... procrustes: rmse 0.06446468 max resid 0.07138561

Run 4 stress 9.405253e-05

... procrustes: rmse 0.1060849 max resid 0.137602

Run 5 stress 0

... procrustes: rmse 0.1224907 max resid 0.1825028

Run 6 stress 0

... procrustes: rmse 0.05581627 max resid 0.06885626

Run 7 stress 0

... procrustes: rmse 0.09636468 max resid 0.1154786

Run 8 stress 0

... procrustes: rmse 0.03045667 max resid 0.03931638

Run 9 stress 0

... procrustes: rmse 0.1385855 max resid 0.185924

Run 10 stress 8.011907e-05

... procrustes: rmse 0.1048179 max resid 0.1388025

Run 11 stress 0

... procrustes: rmse 0.08612398 max resid 0.1371445

Run 12 stress 0

... procrustes: rmse 0.0858111 max resid 0.1211046

Run 13 stress 0

... procrustes: rmse 0.09283316 max resid 0.1572157

Run 14 stress 0

... procrustes: rmse 0.09364433 max resid 0.1302564

Run 15 stress 0

... procrustes: rmse 0.0321095 max resid 0.04822308

Run 16 stress 0

... procrustes: rmse 0.1261124 max resid 0.2018226

Run 17 stress 0

... procrustes: rmse 0.1096793 max resid 0.1419702

Run 18 stress 0

... procrustes: rmse 0.06965235 max resid 0.1085719

Run 19 stress 0

... procrustes: rmse 0.1103221 max resid 0.1353952

Run 20 stress 0

... procrustes: rmse 0.05065781 max resid 0.08095646

Warning message:

In postMDS(out$points, dis, plot = max(0, plot - 1), ...) :

skipping half-change scaling: too few points below threshold

> Elements_5.NMDS.Plot<-ordiplot(Elements_5.NMDS)

> identify(Elements_5.NMDS.Plot, "sites", labels=Elements_ABCD$Site)

warning: no point within 0.25 inches

[1] 1 2 3 4

GREATER THAN OR EQUAL TO 10

> Elements_ABCD[,c(2,3,4,6,8,9,11,12,13,16,18,19,21)]

Astragalus Calcaneum Cranium Femur Humerus Innominate Metapodial Phalanx

Site A 6 6 3 6 6 4 14 7

Site B 4 0 6 15 4 1 14 6

Site C 2 9 7 5 6 3 21 30

Site D 11 4 3 19 12 5 12 27

Podial Scapula Tibia Tooth.Bearing.Elements Vertebra

Site A 2 5 11 77 28

Site B 2 1 8 45 24

Site C 16 2 8 15 39

Site D 10 9 17 16 27

> Elements_10.NMDS<-metaMDS(comm=decostand(Elements_ABCD[,c(2,3,4,6,8,9,11,12,13,16,18,19,21)], "max", distance="bray", k=2, trymax=25, autotransform=F, zerodist="add"))

Run 0 stress 0

Run 1 stress 7.499988e-05

... procrustes: rmse 0.1551102 max resid 0.1888518

Run 2 stress 2.429015e-05

... procrustes: rmse 0.1482017 max resid 0.1883748

Run 3 stress 6.93534e-05

... procrustes: rmse 0.1551083 max resid 0.1888412

Run 4 stress 0

... procrustes: rmse 0.08106564 max resid 0.1063849

Run 5 stress 0

... procrustes: rmse 0.02708698 max resid 0.03585843

Run 6 stress 0

... procrustes: rmse 0.03865378 max resid 0.05396393

Run 7 stress 0

... procrustes: rmse 0.09714852 max resid 0.1549433

Run 8 stress 0

... procrustes: rmse 0.08134948 max resid 0.1197619

Run 9 stress 0

... procrustes: rmse 0.1346041 max resid 0.1714331

Run 10 stress 0

... procrustes: rmse 0.1735745 max resid 0.2562315

Run 11 stress 0

... procrustes: rmse 0.05961064 max resid 0.09422756

Run 12 stress 0.0001036662

... procrustes: rmse 0.1512662 max resid 0.1890156

Run 13 stress 0

... procrustes: rmse 0.1788616 max resid 0.2654574

Run 14 stress 8.022019e-05

... procrustes: rmse 0.1551209 max resid 0.1888675

Run 15 stress 0

... procrustes: rmse 0.181806 max resid 0.2682527

Run 16 stress 0

... procrustes: rmse 0.08357251 max resid 0.1112701

Run 17 stress 7.552582e-05

... procrustes: rmse 0.1551108 max resid 0.188853

Run 18 stress 0

... procrustes: rmse 0.08571937 max resid 0.1377345

Run 19 stress 0

... procrustes: rmse 0.03617413 max resid 0.05377635

Run 20 stress 0

... procrustes: rmse 0.04276985 max resid 0.0645467

Warning message:

In postMDS(out$points, dis, plot = max(0, plot - 1), ...) :

skipping half-change scaling: too few points below threshold

> Elements_10.NMDS.Plot<-ordiplot(Elements_10.NMDS)

> identify(Elements_10.NMDS.Plot, "sites", labels=Elements_ABCD$Site)

[1] 1 2 3 4

GREATER THAN OR EQUAL TO 15

> Elements_ABCD[,c(2,3,4,6,8,11,12,13,16,18,19,21)]

Astragalus Calcaneum Cranium Femur Humerus Metapodial Phalanx Podial

Site A 6 6 3 6 6 14 7 2

Site B 4 0 6 15 4 14 6 2

Site C 2 9 7 5 6 21 30 16

Site D 11 4 3 19 12 12 27 10

Scapula Tibia Tooth.Bearing.Elements Vertebra

Site A 5 11 77 28

Site B 1 8 45 24

Site C 2 8 15 39

Site D 9 17 16 27

> Elements_15.NMDS<-metaMDS(comm=decostand(Elements_ABCD[,c(2,3,4,6,8,11,12,13,16,18,19,21)], "max", distance="bray", k=2, trymax=25, autotransform=F, zerodist="add"))

Run 0 stress 0

Run 1 stress 0

... procrustes: rmse 0.03173383 max resid 0.04841213

Run 2 stress 0.169102

Run 3 stress 0

... procrustes: rmse 0.1031959 max resid 0.1455666

Run 4 stress 0

... procrustes: rmse 0.06303998 max resid 0.07947497

Run 5 stress 6.31035e-05

... procrustes: rmse 0.1206048 max resid 0.1652149

Run 6 stress 0.169102

Run 7 stress 0

... procrustes: rmse 0.1075859 max resid 0.1487135

Run 8 stress 0.169102

Run 9 stress 0

... procrustes: rmse 0.06900242 max resid 0.08905026

Run 10 stress 0

... procrustes: rmse 0.03644773 max resid 0.05832542

Run 11 stress 0

... procrustes: rmse 0.05061612 max resid 0.06512374

Run 12 stress 0

... procrustes: rmse 0.1768703 max resid 0.240751

Run 13 stress 0.169102

Run 14 stress 0

... procrustes: rmse 0.1028721 max resid 0.1558046

Run 15 stress 0

... procrustes: rmse 0.05578946 max resid 0.07307734

Run 16 stress 0

... procrustes: rmse 0.1271951 max resid 0.1496389

Run 17 stress 0.169102

Run 18 stress 0.169102

Run 19 stress 0

... procrustes: rmse 0.05371874 max resid 0.07390208

Run 20 stress 0

... procrustes: rmse 0.1402493 max resid 0.1803333

Warning message:

In postMDS(out$points, dis, plot = max(0, plot - 1), ...) :

skipping half-change scaling: too few points below threshold

> Elements_15.NMDS.Plot<-ordiplot(Elements_15.NMDS)

> identify(Elements_15.NMDS.Plot, "sites", labels=Elements_ABCD$Site)

[1] 1 2 3 4

GREATER THAN OR EQUAL TO 20

> Elements_ABCD[,c(2,6,8,11,12,13,18,19,21)]

Astragalus Femur Humerus Metapodial Phalanx Podial Tibia

Site A 6 6 6 14 7 2 11

Site B 4 15 4 14 6 2 8

Site C 2 5 6 21 30 16 8

Site D 11 19 12 12 27 10 17

Tooth.Bearing.Elements Vertebra

Site A 77 28

Site B 45 24

Site C 15 39

Site D 16 27

> Elements_20.NMDS<-metaMDS(comm=decostand(Elements_ABCD[,c(2,6,8,11,12,13,18,19,21)], "max", distance="bray", k=2, trymax=25, autotransform=F, zerodist="add"))

Run 0 stress 0

Run 1 stress 0.169102

Run 2 stress 0

... procrustes: rmse 0.1171985 max resid 0.173874

Run 3 stress 0.0002611987

... procrustes: rmse 0.1840585 max resid 0.2831433

Run 4 stress 0

... procrustes: rmse 0.3068761 max resid 0.48871

Run 5 stress 0.169102

Run 6 stress 0

... procrustes: rmse 0.2611476 max resid 0.4195859

Run 7 stress 0.0002952336

... procrustes: rmse 0.299653 max resid 0.5002708

Run 8 stress 0.0003484308

... procrustes: rmse 0.1967869 max resid 0.2775936

Run 9 stress 0

... procrustes: rmse 0.1385495 max resid 0.1704371

Run 10 stress 0

... procrustes: rmse 0.2042641 max resid 0.2581493

Run 11 stress 0

... procrustes: rmse 0.1705781 max resid 0.2249598

Run 12 stress 0

... procrustes: rmse 0.1019715 max resid 0.1301116

Run 13 stress 0

... procrustes: rmse 0.1631075 max resid 0.2320068

Run 14 stress 0.169102

Run 15 stress 0

... procrustes: rmse 0.2182615 max resid 0.3219137

Run 16 stress 0

... procrustes: rmse 0.07031423 max resid 0.09155052

Run 17 stress 0.169102

Run 18 stress 0

... procrustes: rmse 0.06596867 max resid 0.09015051

Run 19 stress 0

... procrustes: rmse 0.179982 max resid 0.258405

Run 20 stress 0.0004089589

... procrustes: rmse 0.226185 max resid 0.3580329

Warning message:

In postMDS(out$points, dis, plot = max(0, plot - 1), ...) :

skipping half-change scaling: too few points below threshold

> Elements_20.NMDS.Plot<-ordiplot(Elements_20.NMDS)

> identify(Elements_20.NMDS.Plot, "sites", labels=Elements_ABCD$Site)

[1] 1 2 3 4

GREATER THAN OR EQUAL TO 25

> Elements_ABCD[,c(6,8,11,12,13,18,19,21)]

Femur Humerus Metapodial Phalanx Podial Tibia Tooth.Bearing.Elements

Site A 6 6 14 7 2 11 77

Site B 15 4 14 6 2 8 45

Site C 5 6 21 30 16 8 15

Site D 19 12 12 27 10 17 16

Vertebra

Site A 28

Site B 24

Site C 39

Site D 27

> Elements_25.NMDS<-metaMDS(comm=decostand(Elements_ABCD[,c(6,8,11,12,13,18,19,21)], "max", distance="bray", k=2, trymax=25, autotransform=F, zerodist="add"))

Run 0 stress 0

Run 1 stress 0.169102

Run 2 stress 0

... procrustes: rmse 0.08545239 max resid 0.1186265

Run 3 stress 0

... procrustes: rmse 0.118334 max resid 0.1710426

Run 4 stress 0

... procrustes: rmse 0.1767789 max resid 0.1943945

Run 5 stress 0

... procrustes: rmse 0.02756068 max resid 0.03293167

Run 6 stress 0

... procrustes: rmse 0.09557267 max resid 0.1345351

Run 7 stress 0

... procrustes: rmse 0.1020918 max resid 0.1320332

Run 8 stress 0

... procrustes: rmse 0.07388872 max resid 0.08960574

Run 9 stress 0.169102

Run 10 stress 0.0004502126

... procrustes: rmse 0.2060285 max resid 0.2381913

Run 11 stress 0.169102

Run 12 stress 0.0004212245

... procrustes: rmse 0.2076439 max resid 0.2411105

Run 13 stress 4.525264e-05

... procrustes: rmse 0.2426703 max resid 0.302815

Run 14 stress 0

... procrustes: rmse 0.08251534 max resid 0.1090445

Run 15 stress 0.169102

Run 16 stress 0

... procrustes: rmse 0.1495519 max resid 0.187028

Run 17 stress 0

... procrustes: rmse 0.1136658 max resid 0.1616923

Run 18 stress 0

... procrustes: rmse 0.09067637 max resid 0.1138161

Run 19 stress 0

... procrustes: rmse 0.2378434 max resid 0.3100769

Run 20 stress 0

... procrustes: rmse 0.06082185 max resid 0.08189685

Warning message:

In postMDS(out$points, dis, plot = max(0, plot - 1), ...) :

skipping half-change scaling: too few points below threshold

> Elements_25.NMDS.Plot<-ordiplot(Elements_25.NMDS)

> identify(Elements_25.NMDS.Plot, "sites", labels=Elements_ABCD$Site)

warning: no point within 0.25 inches

[1] 1 2 3 4

GREATER THAN OR EQUAL TO 30

> Elements_ABCD[,c(6,11,12,13,18,19,21)]

Femur Metapodial Phalanx Podial Tibia Tooth.Bearing.Elements Vertebra

Site A 6 14 7 2 11 77 28

Site B 15 14 6 2 8 45 24

Site C 5 21 30 16 8 15 39

Site D 19 12 27 10 17 16 27

> Elements_30.NMDS<-metaMDS(comm=decostand(Elements_ABCD[,c(6,11,12,13,18,19,21)], "max", distance="bray", k=2, trymax=25, autotransform=F, zerodist="add"))

Run 0 stress 0

Run 1 stress 0

... procrustes: rmse 0.1516647 max resid 0.2107167

Run 2 stress 0.169102

Run 3 stress 0.0001151141

... procrustes: rmse 0.1692622 max resid 0.1938377

Run 4 stress 0

... procrustes: rmse 0.04724654 max resid 0.06162425

Run 5 stress 0

... procrustes: rmse 0.08209237 max resid 0.1165728

Run 6 stress 0

... procrustes: rmse 0.1472565 max resid 0.1609019

Run 7 stress 0

... procrustes: rmse 0.1383306 max resid 0.1702825

Run 8 stress 0.001039917

Run 9 stress 0

... procrustes: rmse 0.1443461 max resid 0.2026032

Run 10 stress 0

... procrustes: rmse 0.07584467 max resid 0.1181454

Run 11 stress 0.169102

Run 12 stress 0

... procrustes: rmse 0.05590889 max resid 0.06835368

Run 13 stress 0

... procrustes: rmse 0.1674412 max resid 0.2760768

Run 14 stress 0

... procrustes: rmse 0.08240323 max resid 0.1049171

Run 15 stress 0.0003298546

... procrustes: rmse 0.188003 max resid 0.2003888

Run 16 stress 0.169102

Run 17 stress 0

... procrustes: rmse 0.0981321 max resid 0.1503052

Run 18 stress 0.169102

Run 19 stress 0.0003935323

... procrustes: rmse 0.1909646 max resid 0.2047895

Run 20 stress 0.169102

Warning message:

In postMDS(out$points, dis, plot = max(0, plot - 1), ...) :

skipping half-change scaling: too few points below threshold

> Elements_30.NMDS.Plot<-ordiplot(Elements_30.NMDS)

> identify(Elements_30.NMDS.Plot, "sites", labels=Elements_ABCD$Site)

[1] 1 2 3 4

Initial NMDS Analysis of Element Distribution 3/9/14

> Elements_ABCD[,c(2:21)]

Astragalus Calcaneum Cranium Dentary Femur Fibula Humerus Innominate

Site A 6 6 3 0 6 0 6 4

Site B 4 0 6 1 15 0 4 1

Site C 2 9 7 1 5 1 6 3

Site D 11 4 3 0 19 0 12 5

Maxilla Metapodial Phalanx Podial Radius Rib Scapula Sesamoid Tibia

Site A 0 14 7 2 2 1 5 0 11

Site B 0 14 6 2 2 0 1 0 8

Site C 0 21 30 16 1 3 2 1 8

Site D 1 12 27 10 3 1 9 2 17

Tooth.Bearing.Elements Ulna Vertebra

Site A 77 1 28

Site B 45 1 24

Site C 15 2 39

Site D 16 3 27

> Elements_All.NMDS<-metaMDS(comm=decostand(Elements_ABCD[,c(2:21)], "max", distance="bray", k=2, trymax=25, autotransform=F, zerodist="add"))

Run 0 stress 0

Run 1 stress 0

... procrustes: rmse 0.0535778 max resid 0.06450759

Run 2 stress 7.773251e-05

... procrustes: rmse 0.193207 max resid 0.2526644

Run 3 stress 5.905348e-05

... procrustes: rmse 0.1612304 max resid 0.2339706

Run 4 stress 0.169102

Run 5 stress 0

... procrustes: rmse 0.0573616 max resid 0.06496104

Run 6 stress 0

... procrustes: rmse 0.1305119 max resid 0.1910113

Run 7 stress 0

... procrustes: rmse 0.1157404 max resid 0.1587055

Run 8 stress 0

... procrustes: rmse 0.1575029 max resid 0.2266023

Run 9 stress 0

... procrustes: rmse 0.05541791 max resid 0.07399821

Run 10 stress 0

... procrustes: rmse 0.04780773 max resid 0.05727568

Run 11 stress 0

... procrustes: rmse 0.149721 max resid 0.2189968

Run 12 stress 0

... procrustes: rmse 0.1903653 max resid 0.2436338

Run 13 stress 0.169102

Run 14 stress 0

... procrustes: rmse 0.09539533 max resid 0.1261168

Run 15 stress 0

... procrustes: rmse 0.1371032 max resid 0.1951691

Run 16 stress 0

... procrustes: rmse 0.1603634 max resid 0.1837666

Run 17 stress 0.169102

Run 18 stress 0

... procrustes: rmse 0.1338366 max resid 0.1678922

Run 19 stress 0.169102

Run 20 stress 0

... procrustes: rmse 0.08382924 max resid 0.0939028

Warning message:

In postMDS(out$points, dis, plot = max(0, plot - 1), ...) :

skipping half-change scaling: too few points below threshold

> Elements_All.NMDS.Plot<-ordiplot(Elements_All.NMDS)

> identify(Elements_All.NMDS.Plot, "sites", labels=Elements_ABCD$Site)

[1] 1 2 3 4

GREATER THAN OR EQUAL TO 5

> Elements_ABCD[,c(2,3,4,6,8,9,11,12,13,14,16,18,19,20,21)]

Astragalus Calcaneum Cranium Femur Humerus Innominate Metapodial Phalanx

Site A 6 6 3 6 6 4 14 7

Site B 4 0 6 15 4 1 14 6

Site C 2 9 7 5 6 3 21 30

Site D 11 4 3 19 12 5 12 27

Podial Radius Scapula Tibia Tooth.Bearing.Elements Ulna Vertebra

Site A 2 2 5 11 77 1 28

Site B 2 2 1 8 45 1 24

Site C 16 1 2 8 15 2 39

Site D 10 3 9 17 16 3 27

> Elements_5.NMDS<-metaMDS(comm=decostand(Elements_ABCD[,c(2,3,4,6,8,9,11,12,13,14,16,18,19,20,21)], "max", distance="bray", k=2, trymax=25, autotransform=F, zerodist="add"))

Run 0 stress 0

Run 1 stress 0

... procrustes: rmse 0.05346077 max resid 0.08249621

Run 2 stress 0

... procrustes: rmse 0.05149973 max resid 0.08551588

Run 3 stress 0

... procrustes: rmse 0.06446468 max resid 0.07138561

Run 4 stress 9.405253e-05

... procrustes: rmse 0.1060849 max resid 0.137602

Run 5 stress 0

... procrustes: rmse 0.1224907 max resid 0.1825028

Run 6 stress 0

... procrustes: rmse 0.05581627 max resid 0.06885626

Run 7 stress 0

... procrustes: rmse 0.09636468 max resid 0.1154786

Run 8 stress 0

... procrustes: rmse 0.03045667 max resid 0.03931638

Run 9 stress 0

... procrustes: rmse 0.1385855 max resid 0.185924

Run 10 stress 8.011907e-05

... procrustes: rmse 0.1048179 max resid 0.1388025

Run 11 stress 0

... procrustes: rmse 0.08612398 max resid 0.1371445

Run 12 stress 0

... procrustes: rmse 0.0858111 max resid 0.1211046

Run 13 stress 0

... procrustes: rmse 0.09283316 max resid 0.1572157

Run 14 stress 0

... procrustes: rmse 0.09364433 max resid 0.1302564

Run 15 stress 0

... procrustes: rmse 0.0321095 max resid 0.04822308

Run 16 stress 0

... procrustes: rmse 0.1261124 max resid 0.2018226

Run 17 stress 0

... procrustes: rmse 0.1096793 max resid 0.1419702

Run 18 stress 0

... procrustes: rmse 0.06965235 max resid 0.1085719

Run 19 stress 0

... procrustes: rmse 0.1103221 max resid 0.1353952

Run 20 stress 0

... procrustes: rmse 0.05065781 max resid 0.08095646

Warning message:

In postMDS(out$points, dis, plot = max(0, plot - 1), ...) :

skipping half-change scaling: too few points below threshold

> Elements_5.NMDS.Plot<-ordiplot(Elements_5.NMDS)

> identify(Elements_5.NMDS.Plot, "sites", labels=Elements_ABCD$Site)

warning: no point within 0.25 inches

[1] 1 2 3 4

GREATER THAN OR EQUAL TO 10

> Elements_ABCD[,c(2,3,4,6,8,9,11,12,13,16,18,19,21)]

Astragalus Calcaneum Cranium Femur Humerus Innominate Metapodial Phalanx

Site A 6 6 3 6 6 4 14 7

Site B 4 0 6 15 4 1 14 6

Site C 2 9 7 5 6 3 21 30

Site D 11 4 3 19 12 5 12 27

Podial Scapula Tibia Tooth.Bearing.Elements Vertebra

Site A 2 5 11 77 28

Site B 2 1 8 45 24

Site C 16 2 8 15 39

Site D 10 9 17 16 27

> Elements_10.NMDS<-metaMDS(comm=decostand(Elements_ABCD[,c(2,3,4,6,8,9,11,12,13,16,18,19,21)], "max", distance="bray", k=2, trymax=25, autotransform=F, zerodist="add"))

Run 0 stress 0

Run 1 stress 7.499988e-05

... procrustes: rmse 0.1551102 max resid 0.1888518

Run 2 stress 2.429015e-05

... procrustes: rmse 0.1482017 max resid 0.1883748

Run 3 stress 6.93534e-05

... procrustes: rmse 0.1551083 max resid 0.1888412

Run 4 stress 0

... procrustes: rmse 0.08106564 max resid 0.1063849

Run 5 stress 0

... procrustes: rmse 0.02708698 max resid 0.03585843

Run 6 stress 0

... procrustes: rmse 0.03865378 max resid 0.05396393

Run 7 stress 0

... procrustes: rmse 0.09714852 max resid 0.1549433

Run 8 stress 0

... procrustes: rmse 0.08134948 max resid 0.1197619

Run 9 stress 0

... procrustes: rmse 0.1346041 max resid 0.1714331

Run 10 stress 0

... procrustes: rmse 0.1735745 max resid 0.2562315

Run 11 stress 0

... procrustes: rmse 0.05961064 max resid 0.09422756

Run 12 stress 0.0001036662

... procrustes: rmse 0.1512662 max resid 0.1890156

Run 13 stress 0

... procrustes: rmse 0.1788616 max resid 0.2654574

Run 14 stress 8.022019e-05

... procrustes: rmse 0.1551209 max resid 0.1888675

Run 15 stress 0

... procrustes: rmse 0.181806 max resid 0.2682527

Run 16 stress 0

... procrustes: rmse 0.08357251 max resid 0.1112701

Run 17 stress 7.552582e-05

... procrustes: rmse 0.1551108 max resid 0.188853

Run 18 stress 0

... procrustes: rmse 0.08571937 max resid 0.1377345

Run 19 stress 0

... procrustes: rmse 0.03617413 max resid 0.05377635

Run 20 stress 0

... procrustes: rmse 0.04276985 max resid 0.0645467

Warning message:

In postMDS(out$points, dis, plot = max(0, plot - 1), ...) :

skipping half-change scaling: too few points below threshold

> Elements_10.NMDS.Plot<-ordiplot(Elements_10.NMDS)

> identify(Elements_10.NMDS.Plot, "sites", labels=Elements_ABCD$Site)

[1] 1 2 3 4

GREATER THAN OR EQUAL TO 15

> Elements_ABCD[,c(2,3,4,6,8,11,12,13,16,18,19,21)]

Astragalus Calcaneum Cranium Femur Humerus Metapodial Phalanx Podial

Site A 6 6 3 6 6 14 7 2

Site B 4 0 6 15 4 14 6 2

Site C 2 9 7 5 6 21 30 16

Site D 11 4 3 19 12 12 27 10

Scapula Tibia Tooth.Bearing.Elements Vertebra

Site A 5 11 77 28

Site B 1 8 45 24

Site C 2 8 15 39

Site D 9 17 16 27

> Elements_15.NMDS<-metaMDS(comm=decostand(Elements_ABCD[,c(2,3,4,6,8,11,12,13,16,18,19,21)], "max", distance="bray", k=2, trymax=25, autotransform=F, zerodist="add"))

Run 0 stress 0

Run 1 stress 0

... procrustes: rmse 0.03173383 max resid 0.04841213

Run 2 stress 0.169102

Run 3 stress 0

... procrustes: rmse 0.1031959 max resid 0.1455666

Run 4 stress 0

... procrustes: rmse 0.06303998 max resid 0.07947497

Run 5 stress 6.31035e-05

... procrustes: rmse 0.1206048 max resid 0.1652149

Run 6 stress 0.169102

Run 7 stress 0

... procrustes: rmse 0.1075859 max resid 0.1487135

Run 8 stress 0.169102

Run 9 stress 0

... procrustes: rmse 0.06900242 max resid 0.08905026

Run 10 stress 0

... procrustes: rmse 0.03644773 max resid 0.05832542

Run 11 stress 0

... procrustes: rmse 0.05061612 max resid 0.06512374

Run 12 stress 0

... procrustes: rmse 0.1768703 max resid 0.240751

Run 13 stress 0.169102

Run 14 stress 0

... procrustes: rmse 0.1028721 max resid 0.1558046

Run 15 stress 0

... procrustes: rmse 0.05578946 max resid 0.07307734

Run 16 stress 0

... procrustes: rmse 0.1271951 max resid 0.1496389

Run 17 stress 0.169102

Run 18 stress 0.169102

Run 19 stress 0

... procrustes: rmse 0.05371874 max resid 0.07390208

Run 20 stress 0

... procrustes: rmse 0.1402493 max resid 0.1803333

Warning message:

In postMDS(out$points, dis, plot = max(0, plot - 1), ...) :

skipping half-change scaling: too few points below threshold

> Elements_15.NMDS.Plot<-ordiplot(Elements_15.NMDS)

> identify(Elements_15.NMDS.Plot, "sites", labels=Elements_ABCD$Site)

[1] 1 2 3 4

GREATER THAN OR EQUAL TO 20

> Elements_ABCD[,c(2,6,8,11,12,13,18,19,21)]

Astragalus Femur Humerus Metapodial Phalanx Podial Tibia

Site A 6 6 6 14 7 2 11

Site B 4 15 4 14 6 2 8

Site C 2 5 6 21 30 16 8

Site D 11 19 12 12 27 10 17

Tooth.Bearing.Elements Vertebra

Site A 77 28

Site B 45 24

Site C 15 39

Site D 16 27

> Elements_20.NMDS<-metaMDS(comm=decostand(Elements_ABCD[,c(2,6,8,11,12,13,18,19,21)], "max", distance="bray", k=2, trymax=25, autotransform=F, zerodist="add"))

Run 0 stress 0

Run 1 stress 0.169102

Run 2 stress 0

... procrustes: rmse 0.1171985 max resid 0.173874

Run 3 stress 0.0002611987

... procrustes: rmse 0.1840585 max resid 0.2831433

Run 4 stress 0

... procrustes: rmse 0.3068761 max resid 0.48871

Run 5 stress 0.169102

Run 6 stress 0

... procrustes: rmse 0.2611476 max resid 0.4195859

Run 7 stress 0.0002952336

... procrustes: rmse 0.299653 max resid 0.5002708

Run 8 stress 0.0003484308

... procrustes: rmse 0.1967869 max resid 0.2775936

Run 9 stress 0

... procrustes: rmse 0.1385495 max resid 0.1704371

Run 10 stress 0

... procrustes: rmse 0.2042641 max resid 0.2581493

Run 11 stress 0

... procrustes: rmse 0.1705781 max resid 0.2249598

Run 12 stress 0

... procrustes: rmse 0.1019715 max resid 0.1301116

Run 13 stress 0

... procrustes: rmse 0.1631075 max resid 0.2320068

Run 14 stress 0.169102

Run 15 stress 0

... procrustes: rmse 0.2182615 max resid 0.3219137

Run 16 stress 0

... procrustes: rmse 0.07031423 max resid 0.09155052

Run 17 stress 0.169102

Run 18 stress 0

... procrustes: rmse 0.06596867 max resid 0.09015051

Run 19 stress 0

... procrustes: rmse 0.179982 max resid 0.258405

Run 20 stress 0.0004089589

... procrustes: rmse 0.226185 max resid 0.3580329

Warning message:

In postMDS(out$points, dis, plot = max(0, plot - 1), ...) :

skipping half-change scaling: too few points below threshold

> Elements_20.NMDS.Plot<-ordiplot(Elements_20.NMDS)

> identify(Elements_20.NMDS.Plot, "sites", labels=Elements_ABCD$Site)

[1] 1 2 3 4

GREATER THAN OR EQUAL TO 25

> Elements_ABCD[,c(6,8,11,12,13,18,19,21)]

Femur Humerus Metapodial Phalanx Podial Tibia Tooth.Bearing.Elements

Site A 6 6 14 7 2 11 77

Site B 15 4 14 6 2 8 45

Site C 5 6 21 30 16 8 15

Site D 19 12 12 27 10 17 16

Vertebra

Site A 28

Site B 24

Site C 39

Site D 27

> Elements_25.NMDS<-metaMDS(comm=decostand(Elements_ABCD[,c(6,8,11,12,13,18,19,21)], "max", distance="bray", k=2, trymax=25, autotransform=F, zerodist="add"))

Run 0 stress 0

Run 1 stress 0.169102

Run 2 stress 0

... procrustes: rmse 0.08545239 max resid 0.1186265

Run 3 stress 0

... procrustes: rmse 0.118334 max resid 0.1710426

Run 4 stress 0

... procrustes: rmse 0.1767789 max resid 0.1943945

Run 5 stress 0

... procrustes: rmse 0.02756068 max resid 0.03293167

Run 6 stress 0

... procrustes: rmse 0.09557267 max resid 0.1345351

Run 7 stress 0

... procrustes: rmse 0.1020918 max resid 0.1320332

Run 8 stress 0

... procrustes: rmse 0.07388872 max resid 0.08960574

Run 9 stress 0.169102

Run 10 stress 0.0004502126

... procrustes: rmse 0.2060285 max resid 0.2381913

Run 11 stress 0.169102

Run 12 stress 0.0004212245

... procrustes: rmse 0.2076439 max resid 0.2411105

Run 13 stress 4.525264e-05

... procrustes: rmse 0.2426703 max resid 0.302815

Run 14 stress 0

... procrustes: rmse 0.08251534 max resid 0.1090445

Run 15 stress 0.169102

Run 16 stress 0

... procrustes: rmse 0.1495519 max resid 0.187028

Run 17 stress 0

... procrustes: rmse 0.1136658 max resid 0.1616923

Run 18 stress 0

... procrustes: rmse 0.09067637 max resid 0.1138161

Run 19 stress 0

... procrustes: rmse 0.2378434 max resid 0.3100769

Run 20 stress 0

... procrustes: rmse 0.06082185 max resid 0.08189685

Warning message:

In postMDS(out$points, dis, plot = max(0, plot - 1), ...) :

skipping half-change scaling: too few points below threshold

> Elements_25.NMDS.Plot<-ordiplot(Elements_25.NMDS)

> identify(Elements_25.NMDS.Plot, "sites", labels=Elements_ABCD$Site)

warning: no point within 0.25 inches

[1] 1 2 3 4

GREATER THAN OR EQUAL TO 30

> Elements_ABCD[,c(6,11,12,13,18,19,21)]

Femur Metapodial Phalanx Podial Tibia Tooth.Bearing.Elements Vertebra

Site A 6 14 7 2 11 77 28

Site B 15 14 6 2 8 45 24

Site C 5 21 30 16 8 15 39

Site D 19 12 27 10 17 16 27

> Elements_30.NMDS<-metaMDS(comm=decostand(Elements_ABCD[,c(6,11,12,13,18,19,21)], "max", distance="bray", k=2, trymax=25, autotransform=F, zerodist="add"))

Run 0 stress 0

Run 1 stress 0

... procrustes: rmse 0.1516647 max resid 0.2107167

Run 2 stress 0.169102

Run 3 stress 0.0001151141

... procrustes: rmse 0.1692622 max resid 0.1938377

Run 4 stress 0

... procrustes: rmse 0.04724654 max resid 0.06162425

Run 5 stress 0

... procrustes: rmse 0.08209237 max resid 0.1165728

Run 6 stress 0

... procrustes: rmse 0.1472565 max resid 0.1609019

Run 7 stress 0

... procrustes: rmse 0.1383306 max resid 0.1702825

Run 8 stress 0.001039917

Run 9 stress 0

... procrustes: rmse 0.1443461 max resid 0.2026032

Run 10 stress 0

... procrustes: rmse 0.07584467 max resid 0.1181454

Run 11 stress 0.169102

Run 12 stress 0

... procrustes: rmse 0.05590889 max resid 0.06835368

Run 13 stress 0

... procrustes: rmse 0.1674412 max resid 0.2760768

Run 14 stress 0

... procrustes: rmse 0.08240323 max resid 0.1049171

Run 15 stress 0.0003298546

... procrustes: rmse 0.188003 max resid 0.2003888

Run 16 stress 0.169102

Run 17 stress 0

... procrustes: rmse 0.0981321 max resid 0.1503052

Run 18 stress 0.169102

Run 19 stress 0.0003935323

... procrustes: rmse 0.1909646 max resid 0.2047895

Run 20 stress 0.169102

Warning message:

In postMDS(out$points, dis, plot = max(0, plot - 1), ...) :

skipping half-change scaling: too few points below threshold

> Elements_30.NMDS.Plot<-ordiplot(Elements_30.NMDS)

> identify(Elements_30.NMDS.Plot, "sites", labels=Elements_ABCD$Site)

[1] 1 2 3 4

t-test 3/10/14

> t.test(Eff_Species, y=NULL,alternative=c("two.sided"), mu=0.9879571, paired=FALSE, var.equal=TRUE)

One Sample t-test

data: Eff_Species

t = 148.3699, df = 1, p-value = 0.004291

alternative hypothesis: true mean is not equal to 0.9879571

95 percent confidence interval:

9.012201 10.515295

sample estimates:

mean of x

9.763748

> t.test(Diversity, y=NULL, alternative=c("two.sided"), paired=FALSE, var.equal=TRUE, conf.level=0.95)

One Sample t-test

data: Diversity

t = 165.073, df = 1, p-value = 0.003857

alternative hypothesis: true mean is not equal to 0

95 percent confidence interval:

9.012201 10.515295

sample estimates:

mean of x

9.763748

> t.test(Diversity, y=NULL, alternative=c("less"), paired=FALSE, var.equal=TRUE, conf.level=0.95)

One Sample t-test

data: Diversity

t = 165.073, df = 1, p-value = 0.9981

alternative hypothesis: true mean is less than 0

95 percent confidence interval:

-Inf 10.13719

sample estimates:

mean of x

9.763748

> Voorhies

Aeolian Fluvial

Group I 53 70

Group III 132 42

> t.test(Voorhies, y=NULL, alternative=c("less"), paired=FALSE, var.equal=TRUE, conf.level=0.95)

One Sample t-test

data: Voorhies

t = 3.6953, df = 3, p-value = 0.9828

alternative hypothesis: true mean is less than 0

95 percent confidence interval:

-Inf 121.5362

sample estimates:

mean of x

74.25

> t.test(Voorhies, y=NULL, alternative=c("two.sided"), paired=FALSE, var.equal=TRUE, conf.level=0.95)

One Sample t-test

data: Voorhies

t = 3.6953, df = 3, p-value = 0.03439

alternative hypothesis: true mean is not equal to 0

95 percent confidence interval:

10.30507 138.19493

sample estimates:

mean of x

74.25

K-S Test 3/10/14

> ks.test(Elements_AF$Aeolian, Elements_AF$Fluvial, alternative=c("two.sided"), exact=NULL)

Two-sample Kolmogorov-Smirnov test

data: Elements_AF$Aeolian and Elements_AF$Fluvial

D = 0.25, p-value = 0.5596

alternative hypothesis: two-sided

Warning message:

In ks.test(Elements_AF$Aeolian, Elements_AF$Fluvial, alternative = c("two.sided"), :

cannot compute exact p-values with ties

> ks.test(Size_AF$Aeolian, Size_AF$Fluvial, alternative=c("two.sided"), exact=NULL)

Two-sample Kolmogorov-Smirnov test

data: Size_AF$Aeolian and Size_AF$Fluvial

D = 0.1302, p-value = 0.006065

alternative hypothesis: two-sided

Warning message:

In ks.test(Size_AF$Aeolian, Size_AF$Fluvial, alternative = c("two.sided"), :

p-values will be approximate in the presence of ties

> Taxa_AF<-read.csv("Taxa_AF_1.csv")

> ks.test(Taxa_AF$Aeolian, Taxa_AF$Fluvial, exact=NULL)

Two-sample Kolmogorov-Smirnov test

data: Taxa_AF$Aeolian and Taxa_AF$Fluvial

D = 0.3333, p-value = 0.27

alternative hypothesis: two-sided

Warning message:

In ks.test(Taxa_AF$Aeolian, Taxa_AF$Fluvial, exact = NULL) :

cannot compute exact p-values with ties

> Size_Category

Site.A Site.B Site.C Site.D

1 5 0 3 1

2 29 5 2 0

2.5 2 7 0 0

3 48 13 7 26

3.5 0 0 2 1

4 59 84 10 82

5 37 23 6 69

> ks.test(Size_Category$Site.A+Size_Category$Site.B, Size_Category$Site.C+Size_Category$Site.D, alternative=c("two.sided"), exact=NULL)

Two-sample Kolmogorov-Smirnov test

data: Size_Category$Site.A + Size_Category$Site.B and Size_Category$Site.C + Size_Category$Site.D

D = 0.4286, p-value = 0.5412

alternative hypothesis: two-sided

Regression Code 3/9/14

> Site_A_Regression<-lm(X.MAU~Weathering+Abrasion+Size..mm.+Size.Category+Density+Flatness+Columnarity+SA.V, data=Site_A)

> summary(Site_A_Regression)

Call:

lm(formula = X.MAU ~ Weathering + Abrasion + Size..mm. + Size.Category +

Density + Flatness + Columnarity + SA.V, data = Site_A)

Residuals:

Min 1Q Median 3Q Max

-1540.76 -156.57 51.59 210.11 2243.73

Coefficients:

Estimate Std. Error t value Pr(>|t|)

(Intercept) -1.102e+04 4.477e+02 -24.624 < 2e-16 ***

Weathering -3.040e+01 7.434e+01 -0.409 0.68316

Abrasion -1.141e+02 8.224e+01 -1.387 0.16731

Size..mm. 2.913e-01 3.938e+00 0.074 0.94112

Size.Category -4.640e+01 4.951e+01 -0.937 0.35001

Density 6.427e+03 2.742e+02 23.440 < 2e-16 ***

Flatness -1.081e+03 2.497e+02 -4.328 2.60e-05 ***

Columnarity -1.801e+03 2.184e+02 -8.243 4.85e-14 ***

SA.V 2.147e+02 7.687e+01 2.793 0.00584 **

---

Signif. codes: 0 ‘***’ 0.001 ‘**’ 0.01 ‘*’ 0.05 ‘.’ 0.1 ‘ ’ 1

Residual standard error: 508.7 on 166 degrees of freedom

(9 observations deleted due to missingness)

Multiple R-squared: 0.9232, Adjusted R-squared: 0.9195

F-statistic: 249.6 on 8 and 166 DF, p-value: < 2.2e-16

> Site_B_Regression<-lm(X.MAU~Weathering+Abrasion+Size..mm.+Size.Category+Density+Flatness+Columnarity+SA.V, data=Site_B)

> summary(Site_B_Regression)

Call:

lm(formula = X.MAU ~ Weathering + Abrasion + Size..mm. + Size.Category +

Density + Flatness + Columnarity + SA.V, data = Site_B)

Residuals:

Min 1Q Median 3Q Max

-1419.24 -182.74 54.87 196.61 1016.74

Coefficients:

Estimate Std. Error t value Pr(>|t|)

(Intercept) -7792.629 404.663 -19.257 < 2e-16 ***

Weathering 127.203 63.382 2.007 0.047 *

Abrasion -75.047 91.785 -0.818 0.415

Size..mm. -2.026 2.445 -0.829 0.409

Size.Category 3.412 7.362 0.463 0.644

Density 5329.283 250.867 21.243 < 2e-16 ***

Flatness 59.392 353.804 0.168 0.867

Columnarity -1146.464 167.649 -6.838 3.58e-10 ***

SA.V 34.079 61.259 0.556 0.579

---

Signif. codes: 0 ‘***’ 0.001 ‘**’ 0.01 ‘*’ 0.05 ‘.’ 0.1 ‘ ’ 1

Residual standard error: 370.8 on 120 degrees of freedom

(9 observations deleted due to missingness)

Multiple R-squared: 0.9264, Adjusted R-squared: 0.9215

F-statistic: 188.7 on 8 and 120 DF, p-value: < 2.2e-16

> Site_C_Regression<-lm(X.MAU~Weathering+Abrasion+Size..mm.+Size.Category+Density+Flatness+Columnarity+SA.V, data=Site_C)

> summary(Site_C_Regression)

Call:

lm(formula = X.MAU ~ Weathering + Abrasion + Size..mm. + Size.Category +

Density + Flatness + Columnarity + SA.V, data = Site_C)

Residuals:

Min 1Q Median 3Q Max

-177.82 -35.75 -4.92 32.59 333.12

Coefficients:

Estimate Std. Error t value Pr(>|t|)

(Intercept) -878.5891 675.9959 -1.300 0.208487

Weathering 35.6177 40.4360 0.881 0.388866

Abrasion -6.7477 38.1241 -0.177 0.861293

Size..mm. -0.2078 0.8488 -0.245 0.809083

Size.Category -2.3094 27.6955 -0.083 0.934373

Density 1166.6217 185.4409 6.291 3.84e-06 ***

Flatness 1273.0426 764.8885 1.664 0.111632

Columnarity -595.3514 154.5633 -3.852 0.000995 ***

SA.V 143.1258 45.5120 3.145 0.005099 **

---

Signif. codes: 0 ‘***’ 0.001 ‘**’ 0.01 ‘*’ 0.05 ‘.’ 0.1 ‘ ’ 1

Residual standard error: 111.1 on 20 degrees of freedom

(145 observations deleted due to missingness)

Multiple R-squared: 0.9356, Adjusted R-squared: 0.9099

F-statistic: 36.34 on 8 and 20 DF, p-value: 2.915e-10

> Site_D_Regression<-lm(X.MAU~Weathering+Abrasion+Size..mm.+Size.Category+Density+Flatness+Columnarity+SA.V, data=Site_D)

> summary(Site_D_Regression)

Call:

lm(formula = X.MAU ~ Weathering + Abrasion + Size..mm. + Size.Category +

Density + Flatness + Columnarity + SA.V, data = Site_D)

Residuals:

Min 1Q Median 3Q Max

-524.71 -122.13 -3.78 150.66 571.82

Coefficients:

Estimate Std. Error t value Pr(>|t|)

(Intercept) -663.927 231.105 -2.873 0.00461 **

Weathering 50.694 30.938 1.639 0.10323

Abrasion 23.786 30.867 0.771 0.44205

Size..mm. 2.805 1.319 2.126 0.03501 *

Size.Category -37.350 27.060 -1.380 0.16940

Density 873.536 126.176 6.923 9.65e-11 ***

Flatness -234.476 102.693 -2.283 0.02371 *

Columnarity 482.038 95.943 5.024 1.32e-06 ***

SA.V -66.983 38.371 -1.746 0.08275 .

---

Signif. codes: 0 ‘***’ 0.001 ‘**’ 0.01 ‘*’ 0.05 ‘.’ 0.1 ‘ ’ 1

Residual standard error: 228.7 on 163 degrees of freedom

(21 observations deleted due to missingness)

Multiple R-squared: 0.5997, Adjusted R-squared: 0.5801

F-statistic: 30.53 on 8 and 163 DF, p-value: < 2.2e-16

> Site_AB_Regression<-lm(X.MAU~Weathering+Abrasion+Size..mm.+Size.Category+Density+Flatness+Columnarity+SA.V, data=Site_AB)

> summary(Site_AB_Regression)

Call:

lm(formula = X.MAU ~ Weathering + Abrasion + Size..mm. + Size.Category +

Density + Flatness + Columnarity + SA.V, data = Site_AB)

Residuals:

Min 1Q Median 3Q Max

-1332.02 -359.22 66.51 360.42 1970.75

Coefficients:

Estimate Std. Error t value Pr(>|t|)

(Intercept) -9903.5575 303.2889 -32.654 < 2e-16 ***

Weathering 0.4716 52.4357 0.009 0.992830

Abrasion -133.5118 65.6202 -2.035 0.042784 *

Size..mm. -2.7405 2.2330 -1.227 0.220717

Size.Category -19.6976 9.0614 -2.174 0.030515 *

Density 6142.9953 206.9010 29.691 < 2e-16 ***

Flatness -714.3372 213.1009 -3.352 0.000907 ***

Columnarity -1534.1046 154.6525 -9.920 < 2e-16 ***

SA.V 116.7732 55.7668 2.094 0.037117 *

---

Signif. codes: 0 ‘***’ 0.001 ‘**’ 0.01 ‘*’ 0.05 ‘.’ 0.1 ‘ ’ 1

Residual standard error: 505.4 on 295 degrees of freedom

(18 observations deleted due to missingness)

Multiple R-squared: 0.9055, Adjusted R-squared: 0.903

F-statistic: 353.5 on 8 and 295 DF, p-value: < 2.2e-16

> Site_CD_Regression<-lm(X.MAU~Weathering+Abrasion+Size..mm.+Size.Category+Density+Flatness+Columnarity+SA.V, data=Site_CD)

> summary(Site_CD_Regression)

Call:

lm(formula = X.MAU ~ Weathering + Abrasion + Size..mm. + Size.Category +

Density + Flatness + Columnarity + SA.V, data = Site_CD)

Residuals:

Min 1Q Median 3Q Max

-527.15 -109.72 -5.68 150.75 491.79

Coefficients:

Estimate Std. Error t value Pr(>|t|)

(Intercept) -891.1594 207.2210 -4.301 2.71e-05 ***

Weathering 55.9993 27.8624 2.010 0.0458 *

Abrasion 10.5905 28.0048 0.378 0.7057

Size..mm. 0.9084 0.9918 0.916 0.3609

Size.Category -16.5369 22.4705 -0.736 0.4627

Density 889.4773 116.6399 7.626 1.09e-12 ***

Flatness -237.9638 96.2956 -2.471 0.0143 *

Columnarity 355.6395 87.6017 4.060 7.15e-05 ***

SA.V -29.4546 33.5051 -0.879 0.3804

---

Signif. codes: 0 ‘***’ 0.001 ‘**’ 0.01 ‘*’ 0.05 ‘.’ 0.1 ‘ ’ 1

Residual standard error: 228.3 on 192 degrees of freedom

(166 observations deleted due to missingness)

Multiple R-squared: 0.6046, Adjusted R-squared: 0.5882

F-statistic: 36.7 on 8 and 192 DF, p-value: < 2.2e-16

Residuals Testing (3/9/14)

> Site_A_Regression<-lm(X.MAU~Weathering+Abrasion+Size..mm.+Size.Category+Density+Flatness+Columnarity+SA.V, data=Site_A)

> plot(Site_A_Regression$res, ylab="Residuals", main="Index Plot of Residuals Site A")

> Site_A_Regression_X<-model.matrix(Site_A_Regression)

> Jack_A<-rstudent(Site_A_Regression)

> plot(Jack_A, ylab="Jacknife Residuals", main="Jacknife Residuals for Site A")

> Site_B_Regression<-lm(X.MAU~Weathering+Abrasion+Size..mm.+Size.Category+Density+Flatness+Columnarity+SA.V, data=Site_B)

> plot(Site_B_Regression$res, ylab="Residuals", main="Index Plot of Residuals Site B")

> Jack_B<-rstudent(Site_B_Regression)

> plot(Jack_B, ylab="Jacknife Residuals", main="Jacknife Residuals for Site B")

> Site_C_Regression<-lm(X.MAU~Weathering+Abrasion+Size..mm.+Size.Category+Density+Flatness+Columnarity+SA.V, data=Site_C)

> plot(Site_C_Regression$res, ylab="Residuals", main="Index Plot of Residuals Site C")

> Jack_C<-rstudent(Site_C_Regression)

> plot(Jack_C, ylab="Jacknife Residuals", main="Jacknife Residuals for Site C")

> Site_D_Regression<-lm(X.MAU~Weathering+Abrasion+Size..mm.+Size.Category+Density+Flatness+Columnarity+SA.V, data=Site_D)

> plot(Site_D_Regression$res, ylab="Residuals", main="Index Plot of Residuals Site D")

> Jack_D<-rstudent(Site_D_Regression)

> plot(Jack_D, ylab="Jacknife Residuals", main="Jacknife Residuals for Site D")

> Site_AB_Regression<-lm(X.MAU~Weathering+Abrasion+Size..mm.+Size.Category+Density+Flatness+Columnarity+SA.V, data=Site_AB)

> plot(Site_AB_Regression$res, ylab="Residuals", main="Index Plot of Residuals Sites A and B")

> Jack_AB<-rstudent(Site_AB_Regression)

> plot(Jack_AB, ylab="Jacknife Residuals", main="Jacknife Residuals for Sites A and B")

> Site_CD_Regression<-lm(X.MAU~Weathering+Abrasion+Size..mm.+Size.Category+Density+Flatness+Columnarity+SA.V, data=Site_CD)

> plot(Site_CD_Regression$res, ylab="Residuals", main="Index Plot of Residuals Sites C and D")

> Jack_CD<-rstudent(Site_CD_Regression)

> plot(Jack_CD, ylab="Jacknife Residuals", main="Jacknife Residuals for Sites C and D")

Manova Testing (2/22/13)

> test.man.c.4to12<-manova(cbind(data.c[,4], data.c[,5], data.c[,6],data.c[,7],data.c[,8],data.c[,9],data.c[,10],data.c[,11],data.c[,12])~as.factor(data.c$Site))

> summary(test.man.c.4to12)

Df Pillai approx F num Df den Df Pr(>F)

as.factor(data.c$Site) 3 0.54472 12.227 27 1488 < 2.2e-16 ***

Residuals 502

---

Signif. codes: 0 ‘***’ 0.001 ‘**’ 0.01 ‘*’ 0.05 ‘.’ 0.1 ‘ ’ 1

> test.man.c.8to12<-manova(cbind(data.c[,8],data.c[,9],data.c[,10],data.c[,11],data.c[,12])~as.factor(data.c$Site))

> summary(test.man.c.8to12)

Df Pillai approx F num Df den Df Pr(>F)

as.factor(data.c$Site) 3 0.28794 13.993 15 1977 < 2.2e-16 ***

Residuals 661

---

Signif. codes: 0 ‘***’ 0.001 ‘**’ 0.01 ‘*’ 0.05 ‘.’ 0.1 ‘ ’ 1

> test.man.c.8to11<-manova(cbind(data.c[,8],data.c[,9],data.c[,10],data.c[,11])~as.factor(data.c$Site))

> summary(test.man.c.8to11)

Df Pillai approx F num Df den Df Pr(>F)

as.factor(data.c$Site) 3 0.18106 10.598 12 1980 < 2.2e-16 ***

Residuals 661

---

Signif. codes: 0 ‘***’ 0.001 ‘**’ 0.01 ‘*’ 0.05 ‘.’ 0.1 ‘ ’ 1

> data.lae<-read.csv("data.lae.csv")

> test.man.lae.4to12<-manova(cbind(data.lae[,4], data.lae[,5], data.lae[,6],data.lae[,7],data.lae[,8],data.lae[,9],data.lae[,10],data.lae[,11],data.lae[,12])~as.factor(data.lae$Site))

> test.man.lae.8to12<-manova(cbind(data.l[,8],data.lae[,9],data.lae[,10],data.lae[,11],data.lae[,12])~as.factor(data.lae$Site))

> test.man.lae.8to11<-manova(cbind(data.lae[,8],data.lae[,9],data.lae[,10],data.le[,11])~as.factor(data.l$Site))

> summary(test.man.4to12)

Error in summary(test.man.4to12) : object 'test.man.4to12' not found

> summary(test.man.lae.4to12)

Df Pillai approx F num Df den Df Pr(>F)

as.factor(data.l$Site) 1 0.23661 10.125 9 294 1.411e-13 ***

Residuals 302

---

Signif. codes: 0 ‘***’ 0.001 ‘**’ 0.01 ‘*’ 0.05 ‘.’ 0.1 ‘ ’ 1

> summary(test.man.lae.8to12)

Df Pillai approx F num Df den Df Pr(>F)

as.factor(data.l$Site) 1 0.064074 4.1898 5 306 0.001069 **

Residuals 310

---

Signif. codes: 0 ‘***’ 0.001 ‘**’ 0.01 ‘*’ 0.05 ‘.’ 0.1 ‘ ’ 1

> summary(test.man.lae.8to11)

Df Pillai approx F num Df den Df Pr(>F)

as.factor(data.l$Site) 1 0.019905 1.5587 4 307 0.1852

Residuals 310

> data.cae<-read.csv("data.c.ae.csv")

> test.man.cae.4to12<-manova(cbind(data.cae[,4], data.cae[,5], data.cae[,6],data.cae[,7],data.cae[,8],data.cae[,9],data.cae[,10],data.cae[,11],data.cae[,12])~as.factor(data.cae$Site))

> test.man.cae.8to12<-manova(cbind(data.cae[,8],data.cae[,9],data.cae[,10],data.cae[,11],data.cae[,12])~as.factor(data.cae$Site))

> test.man.cae.8to11<-manova(cbind(data.cae[,8],data.cae[,9],data.cae[,10],data.cae[,11])~as.factor(data.cae$Site))

> summary(test.man.cae.4to12)

Df Pillai approx F num Df den Df Pr(>F)

as.factor(data.cae$Site) 1 0.23856 10.269 9 295 8.792e-14 ***

Residuals 303

---

Signif. codes: 0 ‘***’ 0.001 ‘**’ 0.01 ‘*’ 0.05 ‘.’ 0.1 ‘ ’ 1

> summary(test.man.cae.8to12)

Df Pillai approx F num Df den Df Pr(>F)

as.factor(data.cae$Site) 1 0.063875 4.1895 5 307 0.001068 **

Residuals 311

---

Signif. codes: 0 ‘***’ 0.001 ‘**’ 0.01 ‘*’ 0.05 ‘.’ 0.1 ‘ ’ 1

> summary(test.man.cae.8to11)

Df Pillai approx F num Df den Df Pr(>F)

as.factor(data.cae$Site) 1 0.020196 1.5872 4 308 0.1775

Residuals 311

> data.lfl<-read.csv("data.l.fl.csv")

> test.man.lfl.4to12<-manova(cbind(data.lfl[,4], data.lfl[,5], data.lfl[,6],data.lfl[,7],data.lfl[,8],data.lfl[,9],data.lfl[,10],data.lfl[,11],data.lfl[,12])~as.factor(data.lfl$Site))

> test.man.lfl.8to12<-manova(cbind(data.lfl[,8],data.lfl[,9],data.lfl[,10],data.lfl[,11],data.lfl[,12])~as.factor(data.lfl$Site))

> test.man.lfl.8to11<-manova(cbind(data.lfl[,8],data.lfl[,9],data.lfl[,10],data.lfl[,11])~as.factor(data.lfl$Site))

> summary(test.man.lfl.4to12)

Df Pillai approx F num Df den Df Pr(>F)

as.factor(data.lfl$Site) 1 0.24124 6.7475 9 191 2.181e-08 ***

Residuals 199

---

Signif. codes: 0 ‘***’ 0.001 ‘**’ 0.01 ‘*’ 0.05 ‘.’ 0.1 ‘ ’ 1

> summary(test.man.lfl.8to12)

Df Pillai approx F num Df den Df Pr(>F)

as.factor(data.lfl$Site) 1 0.11914 9.387 5 347 2.101e-08 ***

Residuals 351

---

Signif. codes: 0 ‘***’ 0.001 ‘**’ 0.01 ‘*’ 0.05 ‘.’ 0.1 ‘ ’ 1

> summary(test.man.lfl.8to11)

Df Pillai approx F num Df den Df Pr(>F)

as.factor(data.lfl$Site) 1 0.047012 4.2918 4 348 0.002109 **

Residuals 351

---

Signif. codes: 0 ‘***’ 0.001 ‘**’ 0.01 ‘*’ 0.05 ‘.’ 0.1 ‘ ’ 1

> data.cfl<-read.csv("data.c.fl.csv")

> test.man.cfl.4to12<-manova(cbind(data.cfl[,4], data.cfl[,5], data.cfl[,6],data.cfl[,7],data.cfl[,8],data.cfl[,9],data.cfl[,10],data.cfl[,11],data.cfl[,12])~as.factor(data.cfl$Site))

> test.man.cfl.8to12<-manova(cbind(data.cfl[,8],data.cfl[,9],data.cfl[,10],data.cfl[,11],data.cfl[,12])~as.factor(data.cfl$Site))

> test.man.cfl.8to11<-manova(cbind(data.cfl[,8],data.cfl[,9],data.cfl[,10],data.cfl[,11])~as.factor(data.cfl$Site))

> summary(test.man.cfl.4to12)

Df Pillai approx F num Df den Df Pr(>F)

as.factor(data.cfl$Site) 1 0.23561 6.5412 9 191 4.087e-08 ***

Residuals 199

---

Signif. codes: 0 ‘***’ 0.001 ‘**’ 0.01 ‘*’ 0.05 ‘.’ 0.1 ‘ ’ 1

> summary(test.man.cfl.8to12)

Df Pillai approx F num Df den Df Pr(>F)

as.factor(data.cfl$Site) 1 0.11392 8.8969 5 346 5.815e-08 ***

Residuals 350

---

Signif. codes: 0 ‘***’ 0.001 ‘**’ 0.01 ‘*’ 0.05 ‘.’ 0.1 ‘ ’ 1

> summary(test.man.cfl.8to11)

Df Pillai approx F num Df den Df Pr(>F)

as.factor(data.cfl$Site) 1 0.043875 3.9808 4 347 0.003584 **

Residuals 350

---

Signif. codes: 0 ‘***’ 0.001 ‘**’ 0.01 ‘*’ 0.05 ‘.’ 0.1 ‘ ’ 1

> data.l<-read.csv("DataL.csv")

> test.man.l.4to12<-manova(cbind(data.l[,4], data.l[,5], data.l[,6],data.l[,7],data.l[,8],data.l[,9],data.l[,10],data.l[,11],data.l[,12])~as.factor(data.l$Site))

> test.man.l.8to12<-manova(cbind(data.l[,8],data.l[,9],data.l[,10],data.l[,11],data.l[,12])~as.factor(data.l$Site))

> test.man.l.8to11<-manova(cbind(data.l[,8],data.l[,9],data.l[,10],data.l[,11])~as.factor(data.l$Site))

> summary(test.man.4to12)

Error in summary(test.man.4to12) : object 'test.man.4to12' not found

> summary(test.man.l.4to12)

Df Pillai approx F num Df den Df Pr(>F)

as.factor(data.l$Site) 3 0.55057 12.363 27 1485 < 2.2e-16 ***

Residuals 501

---

Signif. codes: 0 ‘***’ 0.001 ‘**’ 0.01 ‘*’ 0.05 ‘.’ 0.1 ‘ ’ 1

> summary(test.man.l.8to12)

Df Pillai approx F num Df den Df Pr(>F)

as.factor(data.l$Site) 3 0.29115 14.166 15 1977 < 2.2e-16 ***

Residuals 661

---

Signif. codes: 0 ‘***’ 0.001 ‘**’ 0.01 ‘*’ 0.05 ‘.’ 0.1 ‘ ’ 1

> summary(test.man.l.8to11)

Df Pillai approx F num Df den Df Pr(>F)

as.factor(data.l$Site) 3 0.18537 10.867 12 1980 < 2.2e-16 ***

Residuals 661

---

Signif. codes: 0 ‘***’ 0.001 ‘**’ 0.01 ‘*’ 0.05 ‘.’ 0.1 ‘ ’ 1

> test.man.laf.4to12<-manova(cbind(data.l[,4], data.l[,5], data.l[,6],data.l[,7],data.l[,8],data.l[,9],data.l[,10],data.l[,11],data.l[,12])~as.factor(data.l$AF))

> test.man.laf.8to12<-manova(cbind(data.l[,8],data.l[,9],data.l[,10],data.l[,11],data.l[,12])~as.factor(data.l$AF))

> test.man.laf.8to11<-manova(cbind(data.l[,8],data.l[,9],data.l[,10],data.l[,11])~as.factor(data.l$AF))

> summary(test.man.laf.4to12)

Df Pillai approx F num Df den Df Pr(>F)

as.factor(data.l$AF) 1 0.25272 18.6 9 495 < 2.2e-16 ***

Residuals 503

---

Signif. codes: 0 ‘***’ 0.001 ‘**’ 0.01 ‘*’ 0.05 ‘.’ 0.1 ‘ ’ 1

> summary(test.man.laf.8to12)

Df Pillai approx F num Df den Df Pr(>F)

as.factor(data.l$AF) 1 0.23617 40.751 5 659 < 2.2e-16 ***

Residuals 663

---

Signif. codes: 0 ‘***’ 0.001 ‘**’ 0.01 ‘*’ 0.05 ‘.’ 0.1 ‘ ’ 1

> summary(test.man.laf.8to11)

Df Pillai approx F num Df den Df Pr(>F)

as.factor(data.l$AF) 1 0.15091 29.325 4 660 < 2.2e-16 ***

Residuals 663

---

Signif. codes: 0 ‘***’ 0.001 ‘**’ 0.01 ‘*’ 0.05 ‘.’ 0.1 ‘ ’ 1

> data.c<-read.csv("DataC.csv")

> test.man.caf.4to12<-manova(cbind(data.c[,4], data.c[,5], data.c[,6],data.c[,7],data.c[,8],data.c[,9],data.c[,10],data.c[,11],data.c[,12])~as.factor(data.c$AF))

> test.man.caf.8to12<-manova(cbind(data.c[,8],data.c[,9],data.c[,10],data.c[,11],data.c[,12])~as.factor(data.c$AF))

> test.man.caf.8to11<-manova(cbind(data.c[,8],data.c[,9],data.c[,10],data.c[,11])~as.factor(data.c$AF))

> summary(test.man.4to12)

Error in summary(test.man.4to12) : object 'test.man.4to12' not found

> summary(test.man.caf.4to12)

Df Pillai approx F num Df den Df Pr(>F)

as.factor(data.c$AF) 1 0.24822 18.197 9 496 < 2.2e-16 ***

Residuals 504

---

Signif. codes: 0 ‘***’ 0.001 ‘**’ 0.01 ‘*’ 0.05 ‘.’ 0.1 ‘ ’ 1

> summary(test.man.caf.8to12)

Df Pillai approx F num Df den Df Pr(>F)

as.factor(data.c$AF) 1 0.23477 40.435 5 659 < 2.2e-16 ***

Residuals 663

---

Signif. codes: 0 ‘***’ 0.001 ‘**’ 0.01 ‘*’ 0.05 ‘.’ 0.1 ‘ ’ 1

> summary(test.man.caf.8to11)

Df Pillai approx F num Df den Df Pr(>F)

as.factor(data.c$AF) 1 0.14817 28.7 4 660 < 2.2e-16 ***

Residuals 663

---

Signif. codes: 0 ‘***’ 0.001 ‘**’ 0.01 ‘*’ 0.05 ‘.’ 0.1 ‘ ’ 1

> test.man.LDAB<-manova(cbind(L.DAB[,4], L.DAB[,5], L.DAB[,6], L.DAB[,7], L.DAB[,8], L.DAB[,9], L.DAB[,10], L.DAB[,11], L.DAB[,12])~as.factor(L.DAB$DAB))

> summary(test.man.LDAB)

Df Pillai approx F num Df den Df Pr(>F)

as.factor(L.DAB$DAB) 1 0.27208 19.354 9 466 < 2.2e-16 ***

Residuals 474

---

Signif. codes: 0 ‘***’ 0.001 ‘**’ 0.01 ‘*’ 0.05 ‘.’ 0.1 ‘ ’ 1

> test.man.LDAB<-manova(cbind(L.DAB[,8], L.DAB[,9], L.DAB[,10], L.DAB[,11], L.DAB[,12])~as.factor(L.DAB$DAB))

> summary(test.man.LDAB)

Df Pillai approx F num Df den Df Pr(>F)

as.factor(L.DAB$DAB) 1 0.1849 22.094 5 487 < 2.2e-16 ***

Residuals 491

---

Signif. codes: 0 ‘***’ 0.001 ‘**’ 0.01 ‘*’ 0.05 ‘.’ 0.1 ‘ ’ 1

> test.man.LDAB<-manova(cbind(L.DAB[,8], L.DAB[,9], L.DAB[,10], L.DAB[,11])~as.factor(L.DAB$DAB))

> summary(test.man.LDAB)

Df Pillai approx F num Df den Df Pr(>F)

as.factor(L.DAB$DAB) 1 0.14645 20.932 4 488 6.095e-16 ***

Residuals 491

---

Signif. codes: 0 ‘***’ 0.001 ‘**’ 0.01 ‘*’ 0.05 ‘.’ 0.1 ‘ ’ 1

> test.man.CDAB<-manova(cbind(C.DAB[,4], C.DAB[,5], C.DAB[,6], C.DAB[,7], C.DAB[,8], C.DAB[,9], C.DAB[,10], C.DAB[,11], C.DAB[,12])~as.factor(C.DAB$DAB))

> summary(test.man.CDAB)

Df Pillai approx F num Df den Df Pr(>F)

as.factor(C.DAB$DAB) 1 0.26407 18.619 9 467 < 2.2e-16 ***

Residuals 475

---

Signif. codes: 0 ‘***’ 0.001 ‘**’ 0.01 ‘*’ 0.05 ‘.’ 0.1 ‘ ’ 1

> test.man.CDAB<-manova(cbind(C.DAB[,8], C.DAB[,9], C.DAB[,10], C.DAB[,11], C.DAB[,12])~as.factor(C.DAB$DAB))

> summary(test.man.CDAB)

Df Pillai approx F num Df den Df Pr(>F)

as.factor(C.DAB$DAB) 1 0.1818 21.642 5 487 < 2.2e-16 ***

Residuals 491

---

Signif. codes: 0 ‘***’ 0.001 ‘**’ 0.01 ‘*’ 0.05 ‘.’ 0.1 ‘ ’ 1

> test.man.CDAB<-manova(cbind(C.DAB[,8], C.DAB[,9], C.DAB[,10], C.DAB[,11])~as.factor(C.DAB$DAB))

> summary(test.man.CDAB)

Df Pillai approx F num Df den Df Pr(>F)

as.factor(C.DAB$DAB) 1 0.14076 19.986 4 488 2.964e-15 ***

Residuals 491

---

Signif. codes: 0 ‘***’ 0.001 ‘**’ 0.01 ‘*’ 0.05 ‘.’ 0.1 ‘ ’ 1

> test.man.LCAB<-manova(cbind(L.CAB[,4], L.CAB[,5], L.CAB[,6], L.CAB[,7], L.CAB[,8], L.CAB[,9], L.CAB[,10], L.CAB[,11], L.CAB[,12])~as.factor(L.CAB$CAB))

> summary(test.man.LCAB)

Df Pillai approx F num Df den Df Pr(>F)

as.factor(L.CAB$CAB) 1 0.28004 13.959 9 323 < 2.2e-16 ***

Residuals 331

---

Signif. codes: 0 ‘***’ 0.001 ‘**’ 0.01 ‘*’ 0.05 ‘.’ 0.1 ‘ ’ 1

> test.man.LCAB<-manova(cbind(L.CAB[,8], L.CAB[,9], L.CAB[,10], L.CAB[,11], L.CAB[,12])~as.factor(L.CAB$CAB))

> summary(test.man.LCAB)

Df Pillai approx F num Df den Df Pr(>F)

as.factor(L.CAB$CAB) 1 0.18984 22.401 5 478 < 2.2e-16 ***

Residuals 482

---

Signif. codes: 0 ‘***’ 0.001 ‘**’ 0.01 ‘*’ 0.05 ‘.’ 0.1 ‘ ’ 1

> test.man.LCAB<-manova(cbind(L.CAB[,8], L.CAB[,9], L.CAB[,10], L.CAB[,11])~as.factor(L.CAB$CAB))

> summary(test.man.LCAB)

Df Pillai approx F num Df den Df Pr(>F)

as.factor(L.CAB$CAB) 1 0.13674 18.969 4 479 1.713e-14 ***

Residuals 482

---

Signif. codes: 0 ‘***’ 0.001 ‘**’ 0.01 ‘*’ 0.05 ‘.’ 0.1 ‘ ’ 1

> test.man.CCAB<-manova(cbind(C.CAB[,4], C.CAB[,5], C.CAB[,6], C.CAB[,7], C.CAB[,8], C.CAB[,9], C.CAB[,10], C.CAB[,11], C.CAB[,12])~as.factor(C.CAB$CAB))

> summary(test.man.CCAB)

Df Pillai approx F num Df den Df Pr(>F)

as.factor(C.CAB$CAB) 1 0.28013 14.009 9 324 < 2.2e-16 ***

Residuals 332

---

Signif. codes: 0 ‘***’ 0.001 ‘**’ 0.01 ‘*’ 0.05 ‘.’ 0.1 ‘ ’ 1

> test.man.CCAB<-manova(cbind(C.CAB[,8], C.CAB[,9], C.CAB[,10], C.CAB[,11], C.CAB[,12])~as.factor(C.CAB$CAB))

> summary(test.man.CCAB)

Df Pillai approx F num Df den Df Pr(>F)

as.factor(C.CAB$CAB) 1 0.1884 22.239 5 479 < 2.2e-16 ***

Residuals 483

---

Signif. codes: 0 ‘***’ 0.001 ‘**’ 0.01 ‘*’ 0.05 ‘.’ 0.1 ‘ ’ 1

> test.man.CCAB<-manova(cbind(C.CAB[,8], C.CAB[,9], C.CAB[,10], C.CAB[,11])~as.factor(C.CAB$CAB))

> summary(test.man.CCAB)

Df Pillai approx F num Df den Df Pr(>F)

as.factor(C.CAB$CAB) 1 0.1353 18.776 4 480 2.36e-14 ***

Residuals 483

---

Signif. codes: 0 ‘***’ 0.001 ‘**’ 0.01 ‘*’ 0.
